# Pembrolizumab, radiotherapy, and an immunomodulatory five-drug cocktail in pretreated patients with persistent, recurrent, or metastatic cervical or endometrial carcinoma: Results of the phase II PRIMMO study

**DOI:** 10.1007/s00262-022-03253-x

**Published:** 2022-08-12

**Authors:** Emiel A. De Jaeghere, Sandra Tuyaerts, An M. T. Van Nuffel, Ann Belmans, Kris Bogaerts, Regina Baiden-Amissah, Lien Lippens, Peter Vuylsteke, Stéphanie Henry, Xuan Bich Trinh, Peter A. van Dam, Sandrine Aspeslagh, Alex De Caluwé, Eline Naert, Diether Lambrechts, An Hendrix, Olivier De Wever, Koen K. Van de Vijver, Frédéric Amant, Katrien Vandecasteele, Hannelore G. Denys

**Affiliations:** 1 Department of Medical Oncology (Route 535), Ghent University Hospital, C. Heymanslaan 10, 9000 Ghent, Belgium; 2 Cancer Research Institute Ghent (CRIG), Ghent, Belgium; 3 Laboratory of Experimental Cancer Research, Department of Human Structure and Repair, Ghent University, Ghent, Belgium; 4 Gynaecologic Oncology, Department of Oncology, KU Leuven, Leuven, Belgium; 5 Leuven Cancer Institute, Leuven, Belgium; 6 Department of Medical Oncology, University Hospital Brussels, Brussels, Belgium; 7 Laboratory for Medical and Molecular Oncology (LMMO), VUB, Brussels, Belgium; 8 Anticancer Fund (ACF), Strombeek-Bever, Belgium; 9 Biostatistics and Statistical Bioinformatics Centre (L-BioStat), KU Leuven, Leuven, Belgium; 10 Department of Hemato-Oncology, Centre Hospitalier Universitaire Université Catholique de Louvain Namur (Sainte-Elisabeth), Namur, Belgium; 11 Department of Gynecologic Oncology and Senology, University Hospital Antwerp, Edegem, Belgium; 12 Multidisciplinary Oncologic Centre Antwerp (MOCA), University Hospital Antwerp, Edegem, Belgium; 13 Center for Oncological Research (CORE), Integrated Personalized and Precision Oncology Network (IPPON), Edegem, Belgium; 14 Department of Radiation Oncology, Jules Bordet Institute, Brussels, Belgium; 15 Department of Radiation Oncology, General Hospital Sint-Maarten, Mechlin, Belgium; 16 VIB–KU Leuven Center for Cancer Biology, Leuven, Belgium; 17 Department of Pathology, Ghent University Hospital, Ghent, Belgium; 18 Center for Gynecologic Oncology Amsterdam (CGOA), Netherlands Cancer Institute and Amsterdam Medical Center, Amsterdam, The Netherlands; 19 Department of Gynecology and Obstetrics, University Hospitals Leuven, Leuven, Belgium; 20 Department of Radiation Oncology, Ghent University Hospital, Ghent, Belgium

**Keywords:** Radioimmunotherapy, Drug therapy, combination, Gynecologic neoplasms, Immunomodulation, Tumor microenvironment

## Abstract

A phase II study (PRIMMO) of patients with pretreated persistent/recurrent/metastatic cervical or endometrial cancer is presented. Patients received an immunomodulatory five-drug cocktail (IDC) consisting of low-dose cyclophosphamide, aspirin, lansoprazole, vitamin D, and curcumin starting 2 weeks before radioimmunotherapy. Pembrolizumab was administered three-weekly from day 15 onwards; one of the tumor lesions was irradiated (8Gyx3) on days 15, 17, and 19. The primary endpoint was the objective response rate per immune-related response criteria (irORR) at week 26 (a lower bound of the 90% confidence interval [CI] of > 10% was considered efficacious). The prespecified 43 patients (cervical, *n* = 18; endometrial, *n* = 25) were enrolled. The irORR was 11.1% (90% CI 2.0–31.0) in cervical cancer and 12.0% (90% CI 3.4–28.2) in endometrial cancer. Median duration of response was not reached in both cohorts. Median interval-censored progression-free survival was 4.1 weeks (95% CI 4.1–25.7) in cervical cancer and 3.6 weeks (95% CI 3.6–15.4) in endometrial cancer; median overall survival was 39.6 weeks (95% CI 15.0–67.0) and 37.4 weeks (95% CI 19.0–50.3), respectively. Grade ≥ 3 treatment-related adverse events were reported in 10 (55.6%) cervical cancer patients and 9 (36.0%) endometrial cancer patients. Health-related quality of life was generally stable over time. Responders had a significantly higher proportion of peripheral T cells when compared to nonresponders (*p* = 0.013). In conclusion, PRIMMO did not meet its primary objective in both cohorts; pembrolizumab, radiotherapy, and an IDC had modest but durable antitumor activity with acceptable but not negligible toxicity.

*Trial registration* ClinicalTrials.gov (identifier NCT03192059) and EudraCT Registry (number 2016-001569-97).

**Supplementary Information:**

The online version contains supplementary material available at 10.1007/s00262-022-03253-x.

## Introduction

The management of patients with persistent/recurrent/metastatic cervical (CC) or endometrial (EC) carcinoma who are not amenable to curative surgery or radiotherapy has presented an unmet clinical need for decades. Platinum-based chemotherapy had been the standard first-line treatment in both tumor types with a median overall survival (OS) no longer than 17 months [1, 2]. In addition, both tumor types had a similar lack of benefit from second-line chemotherapy and targeted therapies, with response rates of < 20% and median progression-free survival (PFS) limited to 2–5 months without OS improvement [3, 4]. Therefore, historically, no standard second-line treatment for persistent/recurrent/metastatic CC or EC existed after failure of platinum-based chemotherapy [3, 4]. Fortunately, immune checkpoint inhibitors (ICIs) have recently changed the second-line treatment paradigm in these tumor types.

Pembrolizumab and dostarlimab, two programmed death-1 (PD-1) inhibitors, have shown compelling antitumor activity (with response rates ranging from 27 to 57%) in pretreated patients with persistent/recurrent/metastatic microsatellite instability-high (MSI-H) or mismatch repair-deficient (dMMR) EC [5, 6], and the association of lenvatinib and pembrolizumab is the global standard of care for their non–MSI-H/non–dMMR counterparts [7]. In the United States of America (USA), pembrolizumab has also been approved for the second-line and later treatment of patients with persistent/recurrent/metastatic CC whose tumors express programmed death-ligand 1 (PD-L1) [8]. Cemiplimab, another PD-1 inhibitor, was the first drug ever to demonstrate a statistically significant and clinically meaningful OS benefit in pretreated patients with persistent/recurrent/metastatic CC, for which it gained regulatory approval in the USA [9]. Recently, ICIs have even moved to the first-line setting in both the USA and Europe in combination with platinum-based chemotherapy for patients with persistent/recurrent/metastatic CC whose tumors express PD-L1 [10].

Nevertheless, the majority of CC or EC patients either are not responsive to ICI monotherapy or do not have durable clinical benefit (Supplemental Table S1), with the notable exception of those with MSI-H/dMMR EC. This minimal efficacy is likely attributed to an immunosuppressive tumor microenvironment (TME). In addition, although the above-mentioned ICI-based combinations produce interesting response rates, they come at the cost of rather high associated toxicity and financial cost in the absence of truly compelling activity. New combination strategies are, therefore, warranted to assess the combination of ICIs with drugs/modalities that could overcome this immunosuppressive TME in a more affordable and less toxic manner [11].

Radiotherapy, in particular, has been identified as an attractive partner in such combinations, not only because of its established safety profile [12], but also because of its multifaceted immunomodulatory effects, such as the release and presentation of tumor-associated antigens [13], the release of danger signals, the activation of dendritic cells [14], the upregulation of cytokines and chemokines [15], T-cell migration into the tumor bed [16], and the normalization of tumor vasculature [17], essentially converting the irradiated tumor into a personalized in situ vaccine. Recent clinical studies, both single-arm and randomized, provided burgeoning evidence that radiotherapy can enhance ICI-induced antitumor immunity [18–20]. In addition to radiotherapy, low-dose cyclophosphamide might trigger antitumor immunity and synergize with ICIs [21]. Its administration has been demonstrated to promote immunogenic cell death [22], deplete or inactivate regulatory T cells [23], and favor the expansion of cluster of differentiation (CD)8^+^ T cells and natural killer cells [24, 25]. Finally, among drugs approved for non-oncological indications, some have been shown to prompt antitumor immune responses which might help tipping the balance towards increased ICI-induced cytotoxicity in an inexpensive manner (summarized in Supplemental Table S2; reported and discussed in detail elsewhere) [22, 26–28].

Therefore, to decrease immunosuppression and enhance T-cell activation in the TME, we conducted a phase II study of pembrolizumab, stereotactic body radiotherapy (SBRT) (8Gyx3), and an immunomodulatory five-drug cocktail (consisting of low-dose cyclophosphamide, aspirin, lansoprazole, vitamin D, and curcumin) to assess the efficacy and toxicity in pretreated patients with persistent/recurrent/metastatic CC or EC who were unselected for tumor biomarker status (PRIMMO).

## Patients and methods

### Study design and patients

PRIMMO was an investigator-initiated, non-randomized, open-label, multicohort, non-comparative, multisite, phase II study with a safety run-in that enrolled patients across three cohorts: two experimental cohorts of CC and EC, and one parallel exploratory cohort of uterine sarcoma, regardless of subtype [28]. The study protocol has been previously published [28]. The study is registered on ClinicalTrials.gov (identifier NCT03192059) and EudraCT Registry (number 2016-001569-97). Herein, the results of the experimental cohorts are reported.

Key eligibility criteria included age ≥ 18 years; histologically confirmed CC or EC; at least two tumor sites (one index lesion amenable to SBRT and at least one measurable lesion as defined by both immune-related response criteria [irRC] and Response Evaluation Criteria in Solid Tumors, version 1.1, [RECIST v1.1] for response assessment); progression during or after one or more lines of standard chemotherapy (but no upper limit of prior therapies); Eastern Cooperative Oncology Group performance status of 0, 1, or 2; and adequate organ function as determined by laboratory assessments. Patients were enrolled regardless of tumor biomarker expression. A list of exclusion criteria is provided in the supplementary material.

### Procedures

**Fig. 1:**
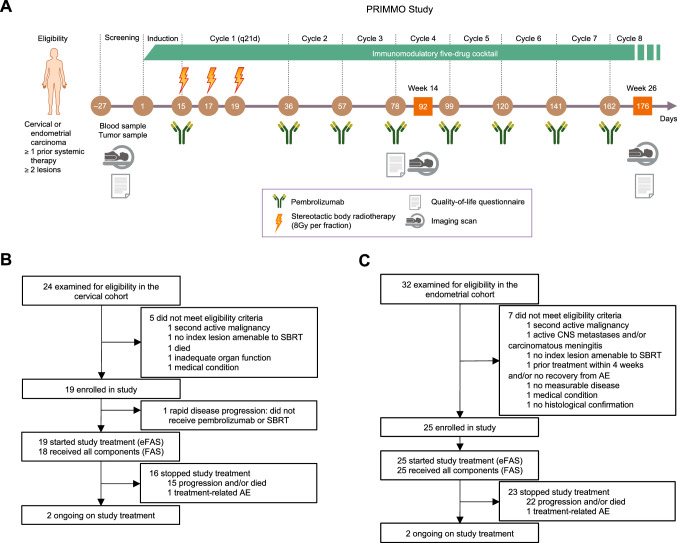
PRIMMO study design. **A** Study procedures. Consolidated Standards of Reporting Trials (CONSORT) diagram of **B** the cervical cohort and **C** the endometrial cohort. *AE* adverse event, *CNS* central nervous system, *(e)FAS* (extended) full analysis set, *SBRT* stereotactic body radiotherapy

Patients received induction with daily intake of 50 mg cyclophosphamide, 325 mg aspirin, 180 mg or 30 mg lansoprazole (dose alternating weekly), 50 μg vitamin D, and 2 g turmeric phytosome (curcumin, a food supplement) for 2 weeks (henceforth dubbed the immunomodulatory five-drug cocktail [IDC]). After this short-term induction period, patients received intravenous pembrolizumab 200 mg once every 3 weeks on an outpatient basis for six cycles or until documented progression, unacceptable toxicity, intercurrent illness preventing additional treatment administration, or voluntary withdrawal from the study. Patients were permitted to remain receiving treatment after progression if they were considered to be benefiting from treatment (at investigator’s discretion). All patients who continued to derive clinical benefit from treatment after six cycles were given the opportunity to continue receiving pembrolizumab for a total of two years within this study. SBRT (24 Gy) was delivered to a single tumor lesion in three fractions over five days during the first cycle of pembrolizumab (study days 15, 17, and 19). The tumor lesion to be irradiated was at the discretion and expertise of the radiation oncologist after consultation with the multidisciplinary team and the patient; a tumor lesion causing symptoms or discomfort to the patient was preferred as target for SBRT. Details of SBRT are provided in the supplementary material. The IDC was administered as maintenance until week 26 but could be continued at investigator’s discretion (Fig. 1A).

Restaging scans were performed at week 14 and week 26; then every 12 weeks thereafter. Unirradiated lesions were measured to assess response to therapy according to irRC and RECIST v1.1 [29–31], both by investigator assessment.

Safety was assessed at least once every 3 weeks until week 26; then every 12 weeks thereafter. Adverse events, hematology and clinical chemistry laboratory values, and vital signs were classified by severity grade according to the National Cancer Institute Common Terminology Criteria for Adverse Events, version 4.0.

Patient-reported HRQOL was evaluated using the Functional Assessment of Cancer Therapy-Cervix (FACT-Cx, version 4.0) questionnaire for the cervical cohort and the FACT-General (FACT-G, version 4.0) questionnaire for the endometrial cohort, completed on-site at baseline, at week 12, week 26, and week 38.

### Study endpoints

The primary endpoint was the objective response rate per irRC (irORR) at week 26, defined as the proportion of patients achieving either complete response (irCR) or partial response (irPR).

Secondary endpoints included ORR at week 26 according to RECIST, version 1.1; best overall response per RECIST v1.1; irPFS, defined as the time from study initiation to first documented progressive disease per irRC or all-cause death, whichever occurred first; OS, defined as the time from study initiation to all-cause death; safety; and patient-reported HRQOL. irPFS and OS were censored at the date of the last follow-up when no event was observed.

Exploratory endpoints are in the supplementary material.

### Immunohistochemical and molecular analysis

All patients were required to provide tumor tissue from a newly obtained core or excisional biopsy sample (preferred) or archival tumor sample of a nonirradiated lesion for central pathology review and translational work by an expert gynecopathologist (KKV) who was blinded for clinical information and patient outcome.

Stromal tumor-infiltrating lymphocytes (sTILs) were assessed with hematoxylin and eosin stained sections, as described by Hendry and colleagues [32]. Tumors were assessed by immunohistochemistry for expression of p16 (as surrogate for human papillomavirus status) and PD-L1 in the cervical cohort, and for expression of hormone receptors, phosphatase and tensin homolog (PTEN), and p53 in the endometrial cohort (Supplemental Table S3). Deoxyribonucleic acid polymerase epsilon exonuclease domain (*POLE*) mutational status and tumor microsatellite status were assessed by next-generation sequencing in the endometrial cohort. Details are provided in the supplementary material (including Supplemental Table S3).

### Peripheral blood mononuclear cell isolation and flow cytometry

Peripheral blood mononuclear cells were isolated from heparinized venous blood (collected at baseline) over a Lymphoprep density gradient (StemCell Technologies, France) and cryopreserved in liquid nitrogen in heat-inactivated human bovine serum supplemented with 10% dimethyl sulfoxide until batch testing. After thawing, the peripheral blood mononuclear cells were stained with fluorescently conjugated mouse antihuman monoclonal antibodies (Supplemental Table S4) for multicolor flow cytometric analysis acquired on BD FACS Canto II cytometer (BD Biosciences, USA) and analyzed with FlowJo software, version 10.6.2 (BD Biosciences, USA). T cells were defined as CD45^+^CD3^+^, helper T cells as CD45^+^CD3^+^CD4^+^CD8^−^, regulatory T cells as CD45^+^CD3^+^CD4^+^CD8^−^CD25^high^CD127^low^FoxP3^+^, and cytotoxic T cells as CD45^+^CD3^+^CD4^−^CD8^+^.

### Systemic inflammatory markers

Total white blood cell count, absolute lymphocyte count, absolute neutrophil count, lactate dehydrogenase, C-reactive protein, and albumin levels were assessed at baseline. The derived neutrophil to lymphocyte ratio, lung immune prognostic index, C-reactive protein to albumin ratio, and modified Glasgow prognostic score were calculated.

### Statistical analysis

The first six patients were included in a safety run-in with real-time reporting of adverse events, dose-limiting toxicity (DLT), and safety analyses. These six patients were assessed and included for all outcomes using the same schedule and criteria as subsequent patients. The study sample size for each cohort was determined using a two-stage design based on exact binomial tests using O’Brien-Fleming stopping boundaries for both efficacy and futility. Sample sizes were determined to achieve approximately 80% power at a one-sided 5% significance level to declare the lower bound of the two-sided 90% CI of the irORR to exceed 10%, assuming an irORR of 35% in the cervical cohort and 30% in the endometrial cohort for study treatment. The planned sample sizes were 18 and 25, respectively. The study was considered to have met its primary objective if the null hypothesis in either cohort was rejected.

Point estimates and exact two-sided 90% confidence intervals (CIs) for binomial proportions were provided for irORR and ORR [33]. Interval-censored irPFS was estimated using the nonparametric Turnbull estimator [34]; right-censored irPFS (post hoc analysis to allow comparison with historic data), OS, and duration of response were estimated with the Kaplan–Meier method [35]. Rates of irPFS and OS were reported along with 95% CIs using the log–log method [35].

To assess safety, descriptive statistics were used to summarize the frequencies of adverse events (all grades and grade ≥ 3). For HRQOL (total and subscales), mean changes from baseline scores were evaluated, as well as (continuous and categorized by clinical benefit) scores over time. Local response rates of irradiated tumor lesions were reported along with 95% CIs using the two-sided Clopper-Pearson exact method.

No comparison between the two cohorts was foreseen. Efficacy, safety, and HRQOL analyses were performed on a full analysis set (patients analyzed who received all study treatment components at least once) and an extended full analysis set (all patients analyzed who started study treatment); the latter is provided in the supplementary material as the primary interest was in the full analysis set (prespecified in the protocol).

Data cutoffs for analysis were June 21, 2019, for the cervical cohort and September 9, 2019, for the endometrial cohort; patients continue to be observed for long-term outcomes. Data analyses were conducted from May 6, 2021, to December 23, 2021. SAS (version 9.4; SAS Institute, Cary, North Carolina, USA) and R (version 4.0.1; R Foundation for Statistical Computing, Vienna, Austria) software were used for statistical analyses.

Key definitions and additional statistical analyses are outlined in the supplementary material.

## Results

### Patient, tumor, and treatment characteristics

Between July 5, 2017, and May 15, 2019, 44 patients (cervical, *n* = 19; endometrial, *n* = 25) were enrolled at four sites in Belgium (Supplemental Table S5). The initially planned interim analyses were not done because of faster than expected accrual into the study. One cervical cancer patient did not receive any pembrolizumab dose due to rapid progression and was not included in the full analysis set as shown in the Consolidated Standards of Reporting Trials diagram (Fig. 1/B/C).

The median follow-up was 36 weeks (interquartile range [IQR], 15–51) in the cervical cohort and 34 weeks (IQR, 15–50) in the endometrial cohort. Patients in both cohorts received a median of four doses of pembrolizumab (IQR; cervical, 3–6; endometrial, 2–6). The median age was 55 years (IQR, 49–63) and 67 years (IQR, 63–71), respectively. The main histological subtype for the cervical cohort was squamous cell carcinoma in 12 (66.7%) patients, whereas for the endometrial cohort it was endometrioid carcinoma in 13 (52.0%) patients. Approximately half of the patients had received one prior systemic therapy for advanced disease (cervical, 7 [38.9%]; endometrial, 15 [60.0%]), whereas the others received two or more. Most patients (11 [61.1%] and 19 [76.0%]) were refractory to their most recent therapy. Table 1 summarizes the demographic and disease characteristics of the patients at baseline. Compliance to SBRT and IDC is reported in Supplemental Table S6 and figure S1, respectively.

**Table 1:** Demographic and disease characteristics of the patients at baseline by disease cohort

	Cervical (*n* = 18)	Endometrial (*n* = 25)
Median follow-up (IQR), weeks	36 (15–51)	34 (15–50)
Median age (IQR), years	55 (49–63)	67 (63–71)
ECOG performance status		
0	10 (55.6)	10 (41.7)
1	7 (38.9)	12 (50.0)
2	1 (5.6)	2 (8.3)
Missing	0	1
FIGO stage at diagnosis		
I–II	9 (52.9)	10 (40.0)
III–IV	8 (47.1)	15 (60.0)
Missing	1	0
Histology		
Cervical		
Squamous cell	12 (66.7)	–
Adenocarcinoma	5 (27.8)	–
Adenosquamous	1 (5.6)	–
Endometrial		
Endometrioid	–	13 (52.0)
Serous	–	11 (44.0)
Clear cell	–	1 (4.0)
Grade		
G1	1 (5.6)	4 (16.0)
G2	4 (22.2)	2 (8.0)
G3	13 (72.2)	19 (76.0)
Prior lines of systemic therapy for advanced disease		
1	7 (38.9)	15 (60.0)
≥ 2	11 (61.1)	10 (40.0)
Prior radiation	11 (61.1)	15 (60.0)
Disease status		
Primary refractory	4 (22.2)	7 (28.0)
Recurrent	7 (38.9)	6 (24.0)
Secondary refractory	7 (38.9)	12 (48.0)
HPV status		
HPV positive	15 (88.2)	–
HPV negative	2 (11.8)	–
Missing	1	–
PD-L1 status (CPS)		
PD-L1 positive	11 (78.6)	–
PD-L1 negative	3 (21.4)	–
Missing	4	–
sTILs		
0–10%	6 (37.5)	14 (66.7)
20–40%	3 (18.8)	5 (23.8)
50–90%	7 (43.8)	2 (9.5)
Missing	2	4
HR status		
HR positive	–	15 (65.2)
HR negative	–	8 (34.8)
Missing	–	2
PTEN status		
Present	–	16 (66.7)
Absent	–	8 (33.3)
Missing	–	1
p53 status		
Wildtype	–	11 (50.0)
Abnormal	–	11 (50.0)
Missing	–	3
Endometrial cancer classification		
Traditional dualistic		
Type I	–	6 (24.0)
Type II	–	19 (76.0)
Histomolecular		
*POLE*mut	–	0
MSI	–	8 (32.0)
p53abn	–	10 (40.0)
NSMP	–	4 (16.0)
NOS	–	3 (12.0)

Data are number of patients (%), unless otherwise indicated

*CPS* combined positivity score, *ECOG* Eastern Cooperative Oncology Group, *IQR* interquartile range, *FIGO* International Federation of Gynecology and Obstetrics, *HPV* human papillomavirus, *HR* hormone receptor, *MSI* microsatellite instability, *NOS* not otherwise specified, *NSMP* non-specific molecular profile, *PD-L1* programmed death-ligand 1, *POLEmut* pathogenic variants in the exonuclease domain of DNA polymerase epsilon, *PTEN* phosphatase and tensin homolog, *sTILs* stromal tumor-infiltrating lymphocytes

### Efficacy

Response per irRC at week 26 was reported in two (11.1%; 90% CI 2.0–31.0) patients in the cervical cohort and in three (12.0%; 90% CI 3.4–28.2) patients in the endometrial cohort. One patient had an irCR (cervical, case ID 6), which is ongoing with 25 doses of pembrolizumab administered thus far; all other responses were irPR. Although one patient (endometrial, case ID 2) experienced marked symptom improvement at week 26, she had received RT to a target lesion for spinal cord compression at week 15 (due to weakening and collapsing bone structures) and was thus not evaluable for response from that moment on; she received 14 doses of pembrolizumab (52 weeks on study) before study discontinuation due to progression. A detailed breakdown of response categories (per irRC and RECIST v1.1) for both cohorts can be found in Table 2.

**Table 2:** Responses by tumor response assessment criteria and by disease cohort

irRC by investigator assessment			RECIST v1.1 by investigator assessment		
Outcome	Cervical (*n* = 18)	Endometrial (*n* = 25)	Outcome	Cervical (*n* = 18)	Endometrial (*n* = 25)
irORR	2^a^ (11.1 [2.0–31.0])	3^a^ (12.0 [3.4–28.2])	ORR	3^a^ (16.7 [4.7–37.7])	3^a^ (12.0 [3.4–28.2])
irCR	1 (5.6 [0.3–23.8])	0 (0 [0–11.3])	CR	1 (5.6 [0.3–23.8])	0 (0 [0–11.3])
irPR	1 (5.6 [0.3–23.8])	3 (12.0 [3.4–28.2])	PR	2 (11.1 [2.0–31.0])	3 (12.0 [3.4–28.2])
irSD	2 (11.1 [2.0–31.0])	0 (0 [0–11.3])	SD	1 (5.6 [0.3–23.8])	0 (0 [0–11.3])
irPD	14 (77.8 [56.1–92.0])	21 (84.0 [67.0–94.3])	PD	14 (77.8 [56.1–92.0])	21 (84.0 [67.0–94.3])
NE	0 (0 [0–15.3])	1 (4.0 [0.2–17.6])	NE	0 (0 [0–15.3])	1 (4.0 [0.2–17.6])
irDCR	4 (22.2 [8.0–43.9])	3 (12.0 [3.4–28.2])	DCR	4 (22.2 [8.0–43.9])	3 (12.0 [3.4–28.2])
–	–^b^	–^b^	BORR	4^c^ (22.2 [8.0–43.9])	3^a^ (12.0 [3.4–28.2])
irDOR (months), median (95% CI)	16.5 + (2.9–16.5 + [NR])	9.0 + (8.2–9.0 + [NR])	DOR (months), median (95% CI)	16.5 + (2.9–16.5 + [NR])	8.7 + (not estimable–8.7 + [NR])

Data are number of patients (% [90% CI]), unless otherwise indicated. ^a^Confirmed responses. ^b^Compared to the patients achieving a response per immune-related response criteria (irRC) at week 26, one additional endometrial cancer patient (case ID 2) and two cervical cancer patients (case IDs 16 and 17) had a partial response per irRC at any point during the study period, although none of these three were confirmed (data not shown in Table because this was not a prespecified endpoint). ^c^One response (case ID 16) was not confirmed

*BORR* best overall response rate, *CR* complete response, *DCR* disease control rate (CR + PR + SD), *DOR* duration of response, *irRC* immune-related response criteria, *NE* not evaluable, *NR* not reached, *ORR* objective response rate, *PD* progressive disease, *PR* partial response, *RECIST v1.1* Response Evaluation Criteria in Solid Tumors version 1.1, *SD* stable disease, *+*  median value was not reached (upper bound corresponds to the longest observed value)

**Fig. 2:**
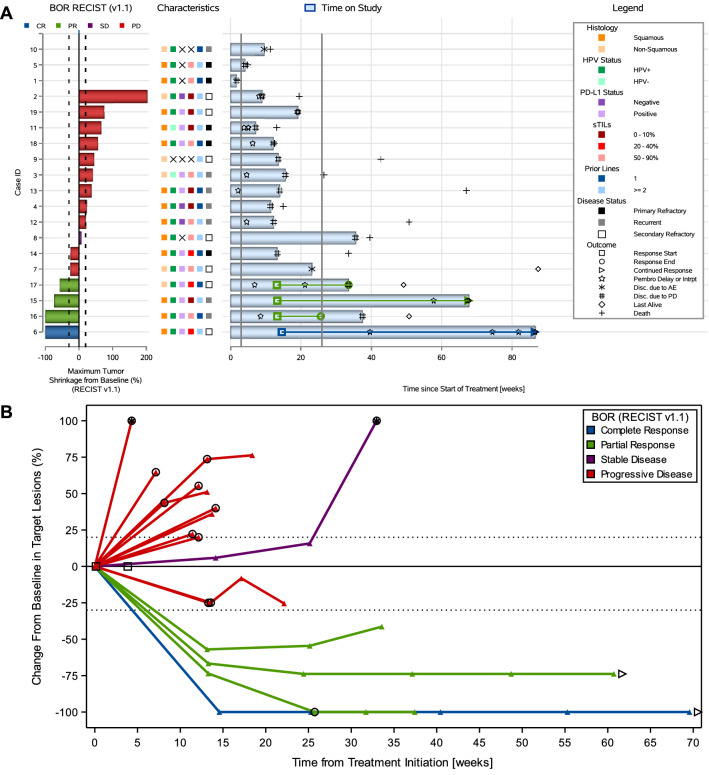

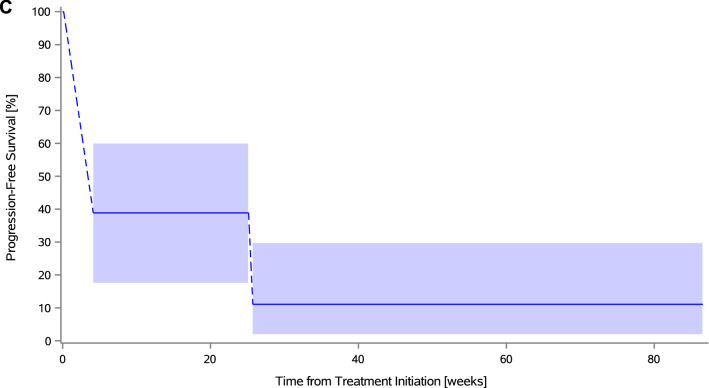
Antitumor activity to study treatment in the cervical cohort. **A** Combined waterfall and swimmer plot. Each row (i.e., response bar + characteristics + swimmer lane) corresponds to one patient. Waterfall plot showing best percentage change from baseline in the sum of diameters of the target lesions; best overall response is indicated by color coding of bars and includes assessment of target, nontarget, and new lesions. The dotted lines at − 30% and + 20% indicate thresholds for partial response and progressive disease (PD), respectively, per Response Evaluation Criteria in Solid Tumors, version 1.1 (RECIST v1.1). Swimmer plot (event chart) for tumor response (response category indicated by color coding) and progressive disease per RECIST v1.1, safety, time on study, and death. The solid lines at week 3 and week 26 indicate the first pembrolizumab dose and the timing of the primary endpoint, respectively. **B** Spider plot. Dynamics of response according to best response (per RECIST v1.1). Circle indicates patients with new lesions or growth in non-target lesions (i.e., PD, even with a less than 20% change in the target lesions). Arrow indicates patients with an ongoing response at time of data cutoff. Square indicates patients with clinical progression (before an imaging scan was acquired). Patients with > 100% increase were truncated at 100% (indicated with a star). **C** Interval-censored progression-free survival per immune-related response criteria. *AE* adverse event, *BOR* best overall response, *CR* complete response, *HPV* human papillomavirus, *PD* progressive disease, *PD-L1* programmed death ligand-1, *PR* partial response, *RECIST v1.1* Response Evaluation Criteria in Solid Tumors, version 1.1, *SD* stable disease, *sTILs* stromal tumor-infiltrating lymphocytes

**Fig. 3:**
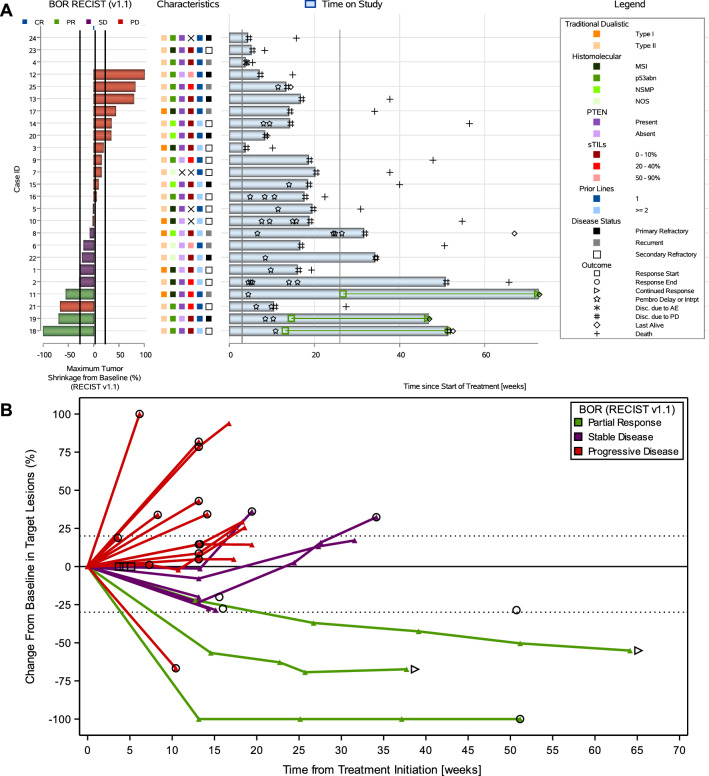

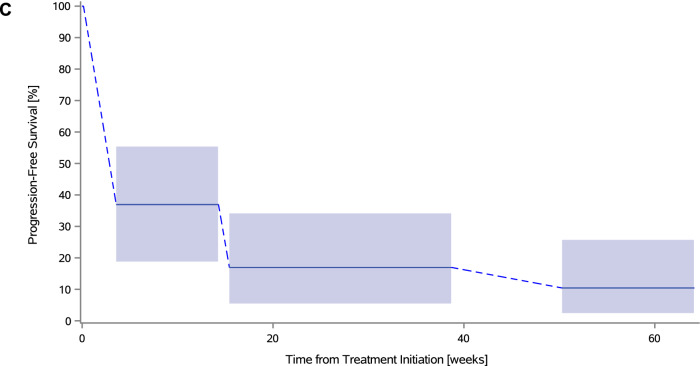
Antitumor activity to study treatment in the endometrial cohort. **A** Combined waterfall and swimmer plot. **B** Spider plot. **C** Interval-censored progression-free survival per immune-related response criteria. *AE* adverse event, *BOR* best overall response, *CR* complete response, *MSI* microsatellite instability, *NOS* not otherwise specified, *NSMP* no specific molecular profile, *PD* progressive disease, *PR* partial response, *PTEN* phosphatase and tensin homolog, *RECIST v1.1* Response Evaluation Criteria in Solid Tumors version 1.1, *SD* stable disease, *sTILs* stromal tumor-infiltrating lymphocytes

All responses per RECIST v1.1 except one (cervical, case ID16) were confirmed by repeat imaging at a subsequent scan at least 4 weeks later; they were generally durable and two of them were ongoing in both cohorts (Figs. 2A and 3A). Decrease in tumor burden was generally maintained over several assessments (Figs. 2B and 3B). All responses were obtained within 26 weeks after study initiation. No pseudoprogression was observed. Only one patient remained on study after first confirmation of progression (Supplemental Table S7).

Median interval-censored irPFS was 4.1 weeks (95% CI 4.1–25.7) in the cervical cohort and 3.6 weeks (95% CI 3.6–15.4) in the endometrial cohort, with a 26-week interval-censored irPFS rate of 11.1% (95% CI 2.8–35.5) and 17.0% (95% CI 6.5–37.8), respectively (Figs. 2C and 3C). Right-censored irPFS data are shown in Supplemental Figure S2. All but 12 patients (six in both cohorts) are known to have died. Median OS was 39.6 weeks (95% CI 15.0–67.0) in the cervical cohort and 37.4 weeks (95% CI 19.0–50.3) in the endometrial cohort (Supplemental Figure S3).

**Fig. 4:**
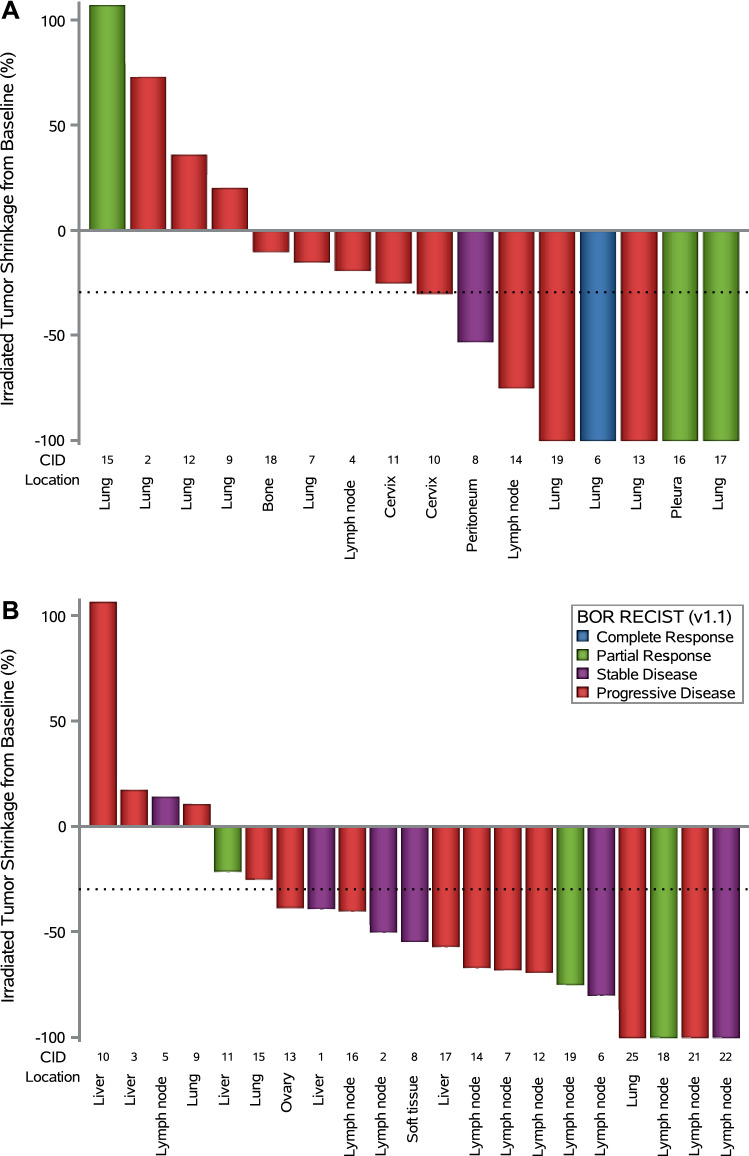
Local response to stereotactic body radiotherapy by disease cohort. **A** Cervical cancer cohort. **B** Endometrial cancer cohort. Bar plot showing the percent change from baseline (at the first imaging scan) in the largest diameter of the irradiated tumor lesion (short axis in case of a nodal lesion); best overall response per Response Evaluation Criteria in Solid Tumors, version 1.1 (RECIST v1.1) is indicated by color coding of bars and includes assessment of target, nontarget, and new lesions (excluding the irradiated tumor lesion). The dotted line at − 30% indicates the threshold for local response. Each column (i.e., response bar + case ID + location of irradiated tumor lesion) corresponds to one patient. *CID* case ID, *RECIST v1.1* Response Evaluation Criteria in Solid Tumors version 1.1

Subgroup analyses did not reveal statistically significant differences in irORR, ORR, interval-censored irPFS, or OS between subgroups, except for sTILs (in ORR, *p* = 0.034) and disease status (in OS, *p* = 0.007) in the cervical cohort (Supplemental Figures S4 and S5). Local response rates of irradiated lesions were 50.0% (95% CI 24.7–75.3) in the cervical cohort and 71.4% (95% CI 47.8–88.7) in the endometrial cohort (Fig. 4).

### Safety

An overview of safety observations is summarized in Table 3. No DLTs occurred during safety run-in. Treatment-related adverse events (TRAEs) of any grade occurred in 16 (88.9%, with 10 [55.6%] grade ≥ 3) and 20 (80.0%, with nine [36%] grade ≥ 3) patients in the cervical and endometrial cohort, respectively. The most common grade 3 or worse TRAEs were colitis (cervical, three [16.7%]; endometrial, three [12.0%]), lymphopenia (three [16.7%] and three [12.0%]), anemia (three [16.7%] and one [4.0%]), and diarrhea (one [5.6%] and three [12.0%]). One patient experienced a TRAE leading to pembrolizumab discontinuation in both cohorts (pulmonary hypertension [5.6%] and colitis [4.0%]). Grade ≥ 3 TRAEs that required pembrolizumab delay or interruption occurred in 5 (27.8%) patients in the cervical cohort and 6 (24.0%) patients in the endometrial cohort. There was one (5.6%) possible treatment-related death in the cervical cohort in a patient who developed pulmonary hypertension soon after the third pembrolizumab dose. Although the patient suffered from intrathoracic progression, treatment relation could not be entirely excluded due to rare reports on (pulmonary) vascular changes after ICI exposure [36]. All other deaths were attributable to progression (11 [61.1%] and 19 [76.0%]).

**Table 3:** Adverse event summary by disease cohort and by severity

Event	Cervical (*n* = 18)		Endometrial (*n* = 25)		All (*n* = 43)	
	Any *G*	*G* ≥ 3	Any *G*	*G* ≥ 3	Any *G*	*G* ≥ 3
Serious TEAE	15 (83.3)		15 (60.0)		30 (69.8)	
Any TRAE	16 (88.9)	10 (55.6)	20 (80.0)	9 (36.0)	36 (83.7)	19 (44.2)
Serious TRAE	9 (50.0)		10 (40.0)		19 (44.2)	
TRAE leading to discontinuation of any treatment component	3 (16.7)	2 (11.1)	3 (12.0)	1 (4.0)	6 (14.0)	3 (7.0)
TRAE leading to delay, interruption, or modification of any treatment component	10 (55.6)	6 (33.3)	16 (64.0)	6 (24.0)	26 (60.5)	12 (27.9)
TRAE leading to discontinuation of pembrolizumab	1 (5.6)	1 (5.6)	1 (4.0)	0	2 (4.7)	1 (2.3)
TRAE leading to delay or interruption of pembrolizumab	8 (44.4)	5 (27.8)	13 (52.0)	6 (24.0)	21 (48.8)	11 (25.6)
TRAE leading to death	1 (5.6)	1 (5.6)	0	0	1 (2.3)	1 (2.3)
TRAE occurring in > 15% of patients in either cohort						
Clinical						
Diarrhea	6 (33.3)	1 (5.6)	11 (44.0)	3 (12.0)	17 (39.5)	4 (9.3)
Fatigue	7 (38.9)	1 (5.6)	8 (32.0)	0	15 (34.9)	1 (2.3)
Colitis	3 (16.7)	3 (16.7)	8 (32.0)	3 (12.0)	11 (25.6)	6 (14.0)
Anorexia	2 (11.1)	0	6 (24.0)	0	8 (18.6)	0
Nausea	2 (11.1)	0	6 (24.0)	0	8 (18.6)	0
Vomiting	2 (11.1)	0	3 (12.0)	1 (4.0)	5 (11.6)	1 (2.3)
Constipation	3 (16.7)	0	4 (16.0)	0	7 (16.3)	0
Rash, maculo-papular	3 (16.7)	1 (5.6)	0	0	3 (7.0)	1 (2.3)
Laboratory						
Anemia	5 (27.8)	3 (16.7)	5 (20.0)	1 (4.0)	10 (23.3)	4 (9.3)
Lymphopenia	4 (22.2)	3 (16.7)	4 (16.0)	3 (12.0)	8 (18.6)	6 (14.0)

Data are number of patients (%) with at least one event. Definitions are outlined in the supplementary material

*TEAE* treatment-emergent adverse event, *TRAE* treatment-related adverse event

### Health-related quality of life

Compliance with the HRQOL questionnaires between baseline and week 26 was at least 80.0% in the cervical cohort and 66.7% in the endometrial cohort (Supplemental Table S8). The continuous HRQOL scores over time are displayed in Supplemental Figure S4. Throughout the 38-week assessment period, HRQOL scores were generally stable in both cohorts. The categorized HRQOL scores at 12, 26, and 38 weeks are displayed in Supplemental Table S9.

### Peripheral blood mononuclear cells

**Fig. 5:**
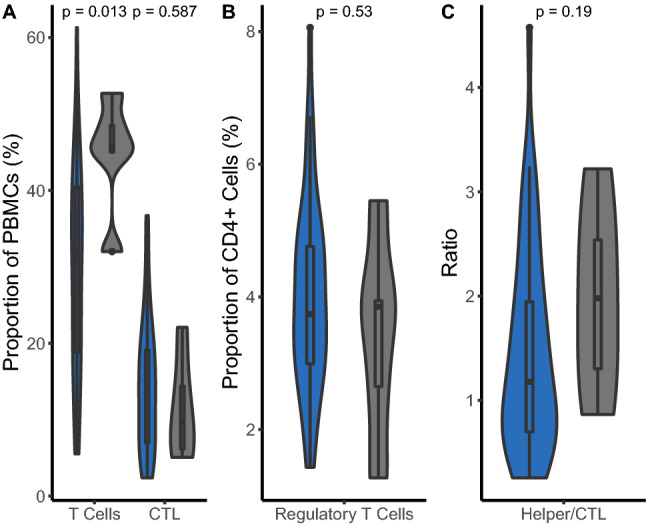
Differences in peripheral T cells between responders and nonresponders. Violin plot showing differences between responders (gray) and nonresponders (blue) in the proportion of peripheral **A** T cells, cytotoxic T cells, and **B** regulatory T cells, as well as in **C** the ratio helper to cytotoxic T cells. Patients were grouped according to their week 26 response per immune-related response criteria (irRC). The *p* values were obtained by the Mann–Whitney U/Wilcoxon Rank-Sum test. *CR* complete response, *CTL* cytotoxic T lymphocyte, *irRC* immune-related response criteria, *PBMC* peripheral blood mononuclear cell, *PD* progressive disease, *PR* partial response, *SD* stable disease

Overall, 42 out of 43 (97.7%) baseline blood samples were available for immunophenotyping of T cells. Responders had a statistically significant higher proportion of peripheral T cells when compared to nonresponders in bivariate analysis (*p* = 0.013). No statistically significant differences between responders and nonresponders were observed based on the proportion of any specific T-cell type (cytotoxic T cells, *p* = 0.587; regulatory T cells, *p* = 0.530; and ratio of helper to cytotoxic T cells, *p* = 0.190) (Fig. 5).

### Systemic inflammatory markers

All four systemic inflammatory markers were available in 40 out of 43 patients (93.0%). There were no statistically significant differences between responders and nonresponders in bivariate analyses (Supplemental Figure S7).

## Discussion

PRIMMO showed that pembrolizumab, SBRT, and an IDC produced a response in approximately 11–17% (depending on the criteria) of patients with persistent/recurrent/metastatic CC and EC, who had at least one previous line of chemotherapy. Whereas the study did not achieve its primary objective, predefined as an irORR with the lower bound of the 90% CI of > 10% in either cohort, and the data from this study are less impressive compared with results observed using other combinations (e.g., nivolumab/ipilimumab in CC and pembrolizumab/lenvatinib in EC) [7, 37], other endpoints, such as the early and durable responses and the stable HRQOL suggest benefits of this treatment in some patients. Despite inherent limitations of cross-study comparison, the observed response rates of this study are similar to those noted for single-agent anti–PD-(L)1. However, given that most patients (69.8%) were refractory to their most recent treatment, a setting marked by increased aggressiveness and resistance to single-agent ICI [38], this should be also appropriately considered when interpreting our results. Furthermore, many patients had other characteristics associated with a lower probability of response to single-agent ICI, such as non-squamous histology (33.3%) in the cervical cohort and p53abn (40.0%) in the endometrial cohort.

The literature on combined ICI and radiotherapy in CC and EC is scarce. In a phase I study (GOG-9929) of 21 patients with node-positive locally advanced CC, the use of ipilimumab sequentially after chemoradiotherapy has been shown to be safe and feasible (any grade, not reported; grade ≥ 3, 10%) [39]. A two-arm phase I study showed no apparent improvement to the response rate from adding radiotherapy (9Gyx3) to cemiplimab treatment versus single-agent cemiplimab (one PR in ten patients [10%] in both arms) in persistent/recurrent/metastatic CC patients who were resistant to or intolerant of platinum and taxane chemotherapy [40]. Both studies were not designed or powered to assess efficacy. To our knowledge, the combination of ICI and radiotherapy has not yet been investigated in EC. In the studies that evaluated the combination of ICI and radiotherapy among patients with other solid tumors, response rates varied widely. For instance, Luke et al*.* reported a modest 13% response rate in a phase I study of SBRT (dose varied by anatomic site) to two to four metastases followed by pembrolizumab in heavily pretreated patients with a variety of primary cancers [18], while Hammers, et al. reported an encouraging response rate of 56% in patients with metastatic clear cell renal cell carcinoma receiving dual anti-PD-1/cytotoxic T-lymphocyte-associated protein 4 with nivolumab/ipilimumab and concurrent, higher dose SBRT (10Gyx5) to only one or two metastases [19]. The reasons for the overall null findings in the present study are unclear but differences in technical aspects of treatment such as total dose, fractionation, dose heterogeneity, target site(s), volume of radiation (e.g., ablation of single metastasis, all, or as many as possible), and optimal sequencing in relation to ICI among different studies are likely to underlie the contradictory results [41]. Such radiotherapy differences could result in distinct immunomodulatory effects. Alternatively, a more nuanced explanation may relate to the heterogenous groups of patients under study or differences in tumor burden, tumor spread (oligometastatic or polymetastatic), total treatment duration, and type of ICI. While we recognize that significant work has been done to explain radiotherapy’s immunological impact, these and our data suggest that a more thorough understanding is needed to identify the radiotherapy schedule required to achieve an optimal immune response. Therefore, the widely adopted ‘one-size-fits-all’ strategy of 8Gyx3 is not always the optimal choice to combine with ICI, and both patient-specific and tumor-specific characteristics should determine whether and how radiotherapy should be combined with ICI.

Non-commercial repurposing of generic or off-patent drugs has increasingly become recognized as a cost-efficient way to develop new, widely available, and affordable cancer treatments [26]. Similar to our IDC (and radiotherapy) strategy, Herrera, et al*.* recently reported on a combined preclinical and phase I clinical study demonstrating that nivolumab, ipilimumab, low-dose radiotherapy, low-dose cyclophosphamide, and CD40ag/aspirin all contributed to a profound reprogramming of the TME in immune desert tumors. Although these results were widely appreciated as positive, the reported response rate (one PR in eight patients [12.5%]) was relatively low and comparable to that reported here [16]. The scientific rationale supporting our IDC originates from many sources mentioned in more detail in Supplemental Table S2 [28]. Despite the promising preliminary evidence, our results suggest that further study is warranted to translate this biological potential into clinical practice. One explanation for our lower than anticipated efficacy is that a fourth of patients experienced rapid progression and received only one or two pembrolizumab doses, which may reflect the aggressive biology and poor prognosis of non-immunoreactive tumors with escape mechanisms bypassing the PD-1/PD-L1 axis as well as the targeted immunomodulatory pathways [42]. Indeed, about half of our patients had a tumor with an immune desert phenotype characterized by scarce or absent sTILs. Another explanation is that we cannot exclude a negative impact of the IDC leading to accelerated tumor growth. For instance, recent studies across a broad variety of cancers have suggested that proton pump inhibitors could negatively affect outcomes in ICI-treated patients [43].

The observed toxicity profile was less favorable than what has previously been reported with combined PD-1 inhibitors and radiotherapy in other tumor types, although these studies cannot be compared in a formal manner [44]. Patients in both cohorts experienced frequent (any grade, 83.7%; grade ≥ 3, 44.2%), but not unexpected, TRAEs consisting mainly of mild to moderate gastrointestinal toxicities and fatigue. Although no DLTs were noted within the 7-week safety run-in period, grade ≥ 3 TRAEs occurring beyond this window were rather frequent. In particular, grade ≥ 3 colitis affected 14% of patients. Possible explanations for this are that patients may have developed aspirin-mediated intestinal epithelial dysfunction [45], had their gut microbiome disrupted by the IDC [46], and often underwent pelvic surgery and/or radiotherapy. In addition, grade ≥ 3 anemia and lymphopenia were observed in 9.3% and 14.0% of patients, respectively. This is higher than what would be expected (< 5%) [47], a result with no clear explanation of the mechanism. It is important to note that the higher incidence of grade ≥ 3 TRAEs was not reflected in a higher pembrolizumab discontinuation rate (4.7%). This may, however, be due to the limited drug exposure of the subgroup of patients who experienced rapid progression. Nonetheless, our results suggest that the study treatment had little adverse impact on HRQOL.

Subgroup analyses showed no consistent pattern of benefit with study treatment, including not in PD-L1–positive (cervical cohort) or MSI-H (endometrial cohort) tumors, although these were neither powered nor corrected for multiple comparisons and should be interpreted with caution. Particular caution should be warranted due to our very small number of MSI-H tumors (*n* = 8) and wide confidence intervals. Similarly, the presented translational work should be interpreted as exploratory. Nonetheless, our results suggest that peripheral T cells could be a valuable marker of response to the study treatment.

There are limitations to this study. The main limitations include the small number of patients in each disease cohort and the lack of a randomly allocated control group, combined with the broad historic response rates to single-agent anti-PD-1 in both diseases (0–57%, depending on biomarker profiles), which became apparent during the conduct of this study. Second, although the concurrent assessment of seven therapy components allowed parallel focus on multiple immunomodulatory mechanisms, incremental stepwise assessment would have made it easier to unveil the individual contributions of the components. Because of the clinical pressure for achieving response in the studied populations and the lower overall costs we favored the concurrent assessment. Third, tumor response assessments were not independently reviewed.

In conclusion, the combination of pembrolizumab, SBRT, and an IDC was justified by preclinical evidence but did not meet expectations of clinical activity in both cohorts; however, some patients may have derived benefit from treatment, with durable responses in difficult-to-treat patients. It is therefore worth to further investigate ICIs, either alone in biomarker-enriched populations or in novel combinations in persistent/recurrent/metastatic CC or EC, as evidenced by recent successes [5–7, 10].

## Supplementary Information

Below is the link to the electronic supplementary material.

Supplementary file1 (DOCX 430 KB)

## Data Availability

The datasets used and/or analyzed during the current study are available from the corresponding author on reasonable request.

## Abbreviations

CC: Cervical carcinoma
CD: Cluster of differentiation
CI: Confidence interval
CR: Complete response
DLT: Dose-limiting toxicity
dMMR: Mismatch repair-deficient
EC: Endometrial carcinoma
FACT: Functional Assessment of Cancer Therapy
HRQOL: Health-related quality of life
ICI: Immune checkpoint inhibitor
IDC: Immunomodulatory five-drug cocktail
IQR: Interquartile range
ir- prefix: Indication that the immune-related response criteria (irRC) were used
irRC: Immune-related response criteria
MSI-H: Microsatellite instability-high
ORR: Objective response rate
OS: Overall survival
PD-(L)1: Programmed death-(ligand) 1
PFS: Progression-free survival
*POLE*: Deoxyribonucleic acid polymerase epsilon (exonuclease domain)
PR: Partial response
PTEN: Phosphatase and tensin homolog
RECIST: Response Evaluation Criteria in Solid Tumors
SBRT: Stereotactic body radiotherapy
sTILs: Stromal tumor-infiltrating lymphocytes
TME: Tumor microenvironment
TRAE: Treatment-related adverse event
USA: United States of America
