# Correction: Pembrolizumab, radiotherapy, and an immunomodulatory five-drug cocktail in pretreated patients with persistent, recurrent, or metastatic cervical or endometrial carcinoma: Results of the phase II PRIMMO study

**DOI:** 10.1007/s00262-024-03873-5

**Published:** 2024-12-30

**Authors:** Emiel A. De Jaeghere, Sandra Tuyaerts, An M. T. Van Nufel, Ann Belmans, Kris Bogaerts, Regina Baiden-Amissah, Lien Lippens, Peter Vuylsteke, Stéphanie Henry, Xuan Bich Trinh, Peter A. van Dam, Sandrine Aspeslagh, Alex De Caluwé, Eline Naert, Diether Lambrechts, An Hendrix, Olivier De Wever, Koen K. Van de Vijver, Frédéric Amant, Katrien Vandecasteele, Hannelore G. Denys

**Affiliations:** 1 Department of Medical Oncology (Route 535), Ghent University Hospital, C. Heymanslaan 10, 9000 Ghent, Belgium; 2 Cancer Research Institute Ghent (CRIG), Ghent, Belgium; 3 Laboratory of Experimental Cancer Research, Department of Human Structure and Repair, Ghent University, Ghent, Belgium; 4 Gynaecologic Oncology, Department of Oncology, KU Leuven, Louvain, Belgium; 5 Leuven Cancer Institute, Louvain, Belgium; 6 Department of Medical Oncology, University Hospital Brussels, Brussels, Belgium; 7 Laboratory for Medical and Molecular Oncology (LMMO), VUB, Brussels, Belgium; 8 Anticancer Fund (ACF), Strombeek-Bever, Belgium; 9 Biostatistics and Statistical Bioinformatics Centre (L-BioStat), KU Leuven, Louvain, Belgium; 10 Department of Hemato-Oncology, Centre Hospitalier Universitaire Université Catholique de Louvain Namur (Sainte-Elisabeth), Namur, Belgium; 11 Department of Gynecologic Oncology and Senology, University Hospital Antwerp, Edegem, Belgium; 12 Multidisciplinary Oncologic Centre Antwerp (MOCA), University Hospital Antwerp, Edegem, Belgium; 13 Center for Oncological Research (CORE), Integrated Personalized and Precision Oncology Network (IPPON), Edegem, Belgium; 14 Department of Radiation Oncology, Jules Bordet Institute, Brussels, Belgium; 15 Department of Radiation Oncology, General Hospital Sint-Maarten, Mechlin, Belgium; 16 VIB–KU Louvain Center for Cancer Biology, Leuven, Belgium; 17 Department of Pathology, Ghent University Hospital, Ghent, Belgium; 18 Center for Gynecologic Oncology Amsterdam (CGOA), Netherlands Cancer Institute and Amsterdam Medical Center, Amsterdam, The Netherlands; 19 Department of Gynecology and Obstetrics, University Hospitals Leuven, Louvain, Belgium; 20 Department of Radiation Oncology, Ghent University Hospital, Ghent, Belgium

**Correction to: Cancer Immunology, Immunotherapy (2022) 72:475–491** 10.1007/s00262-022-03253-x

In the original version of this article, there is a minor typo in the third sentence of the introduction section where "USA" should be "EU".

The third sentence of the introduction section, which previously read

"Cemiplimab, another PD-1 inhibitor, was the first drug ever to demonstrate a statistically significant and clinically meaningful OS benefit in pretreated patients with persistent/recurrent/metastatic CC, for which it gained regulatory approval in the USA".

Should have read

"Cemiplimab, another PD-1 inhibitor, was the first drug ever to demonstrate a statistically significant and clinically meaningful OS benefit in pretreated patients with persistent/recurrent/metastatic CC, for which it gained regulatory approval in the EU".

The vertical dotted lines in Fig. 3A (the waterfall plot) were incorrectly depicted as a solid line. The Fig. 3 should have appeared as shown below.

**Fig. 3:**
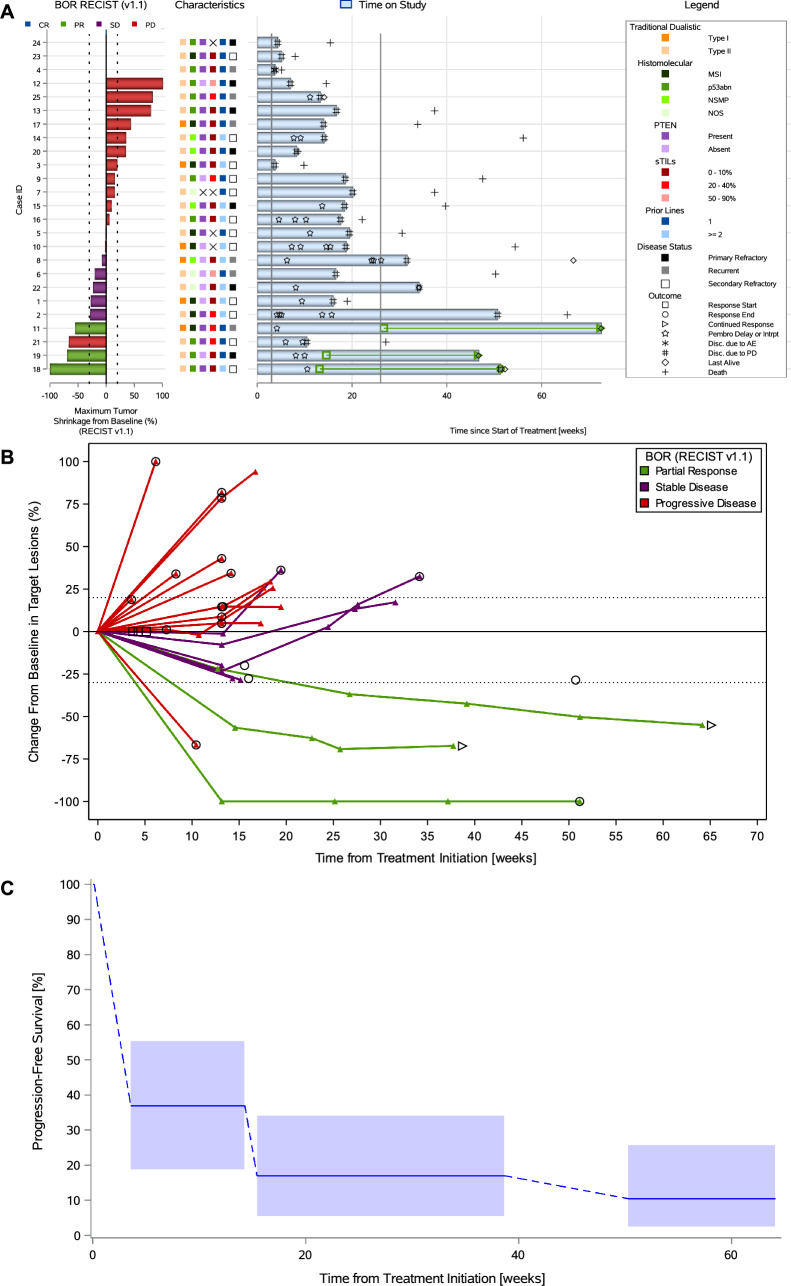
Antitumor activity to study treatment in the endometrial cohort.** A** Combined waterfall and swimmer plot.** B** Spider plot.** C** Interval-censored progression-free survival per immune-related response criteria. *AE* adverse event, *BOR* best overall response,* CR* complete response,* MSI* microsatellite instability,* NOS* not otherwise specified,* NSMP* no specific molecular profile,* PD* progressive disease,* PR* partial response,* PTEN* phosphatase and tensin homolog,* RECIST v1.1* Response Evaluation Criteria in Solid Tumors version 1.1,* SD* stable disease,* sTILs* stromal tumor-infiltrating lymphocytes

