# Supplementary material for: Pembrolizumab, radiotherapy, and an immunomodulatory five-drug cocktail in pretreated patients with persistent, recurrent, or metastatic cervical or endometrial carcinoma: Results of the phase II PRIMMO study
Source: Cancer Immunol Immunother. 2022 Aug 12;72(2):475–91. doi: 10.1007/s00262-022-03253-x (PMC9870976; doi:10.1007/s00262-022-03253-x)
Supplement: Supplementary file 1 — Supplementary file1 (DOCX 430 KB) [file 262_2022_3253_MOESM1_ESM.docx]

**Pembrolizumab, Radiotherapy, and an Immunomodulatory Five-Drug Cocktail in Pretreated Patients with Persistent, Recurrent, or Metastatic Cervical or Endometrial Carcinoma: Results of the Phase II PRIMMO Study**

**Supplementary Material**

[1. List of Abbreviations 2](#_Toc105684241)

[2. Exclusion Criteria 3](#_Toc105684242)

[3. Stereotactic Body Radiotherapy Details 3](#_Toc105684243)

[4. Exploratory Endpoints 4](#_Toc105684244)

[5. Key Definitions 4](#_Toc105684245)

[6. Additional Statistical Analyses 8](#_Toc105684246)

[7. Extended Full Analysis Set of the Cervical Cohort 9](#_Toc105684247)

[8. Supplementary Figures 11](#_Toc105684248)

[9. Supplementary Tables 20](#_Toc105684249)

[10. References 29](#_Toc105684250)

# List of Abbreviations

CAR: C-reactive protein to albumin ratio

CR: complete response

dNLR: derived neutrophil to lymphocyte ratio

EC: endometrial carcinoma

eFAS: extended full analysis set

FACT: Functional Assessment of Cancer Therapy

IHC: immunohistochemistry

ir- prefix: indication that the immune-related response criteria (irRC) were used

irRC: immune-related response criteria

LIPI: lung immune prognostic index

mGPS: modified Glasgow prognostic score

MID: minimal clinically important difference

MSI-H: microsatellite instability-high

NE: not estimable

NOS: not otherwise specified

NSMP: non-specific molecular profile

ORR: objective response rate

PD: progressive disease

PD-(L)1/2: programmed cell death receptor-(ligand) 1/2

PFS: progression-free survival

*POLE*: deoxyribonucleic acid polymerase epsilon (exonuclease domain)

PR: partial response

PTEN: phosphatase and tensin homolog

RECIST: Response Evaluation Criteria in Solid Tumors

SD: stable disease

TEAE: treatment-emergent adverse event

# Exclusion Criteria

Patients were excluded from enrollment if they had received chemotherapy, targeted small-molecule therapy, anticancer monoclonal antibody, endocrine therapy, radiotherapy, or investigational agent in the previous four weeks; active central nervous system metastases (previously treated brain metastases were permitted if stable) or carcinomatous meningitis; received prior therapy with an agent directed against programmed cell death receptor-(ligand) 1 (PD-[L]1) or PD-L2; active autoimmune disease that required systemic treatment in the previous two years, with the exception of replacement therapy (e.g., thyroxine, insulin, or physiologic corticosteroid replacement therapy for adrenal/pituitary insufficiency); diagnosis of immunodeficiency or received systemic steroid therapy or any other form of immunosuppressive in the previous two weeks; active infection requiring systemic therapy; known history of active tuberculosis, human immunodeficiency virus, human T-lymphotropic virus, syphilis, hepatitis B, or hepatitis C; known history of non-infectious pneumonitis; received live vaccine 30 days before initiating study treatment; or adverse events from previous therapy that had not resolved to grade 2 or less, or baseline.

# Stereotactic Body Radiotherapy Details

The gross tumor volume was defined as all visible tumor of the index lesion by combining iconographic and metabolic information (if available). No additional margin was added for microscopic spread of disease. The gross tumor volume was expanded by 2 to 5 mm to the planning target volume to account for organ motion and setup variance. These margins depended on the site irradiated, with 2-mm margins for bony lesions and 5-mm margins for other sites. A total dose of 24Gy (80% of the maximal dose) was delivered in three fractions with each fraction 48 hours apart (Monday, Wednesday, and Friday afternoon). Treatment was prescribed to the periphery of the target (80% of the dose [24Gy]), covering 90% of the planning target volume. At each fraction, a cone-beam computed tomography was used for patient setup and target verification before treatment. Dose constraints of organs at risk were in accordance with the recommendations of the American Association of Physicist in Medicine task group 101 report (1).

# Exploratory Endpoints

Prespecified exploratory endpoints included quantifying baseline peripheral immune cells and systemic inflammatory markers and their association with tumor response. P*ost hoc* exploratory endpoints were the duration of response in patients who had a best response of (ir)PR or (ir)CR, defined as time from initial response to progression per irRC (irDOR) or RECIST v1.1 (DOR), or death from any cause; and the local response rate of irradiated lesions (by disease cohort), defined as a ≥30% decrease in the longest diameter of the irradiated lesion (short axis in case of a nodal lesion).

Other prespecified exploratory analyses (results of the exploratory uterine sarcoma cohort and additional translational work, including [longitudinal] gut microbiome analysis, longitudinal immune monitoring, and baseline TME analysis) are ongoing and will be reported separately.

# Key Definitions

*Patient Characteristics*

Disease status was defined as a categorical variable with three categories: primary refractory (no complete response after frontline therapy and no partial/complete response with any salvage therapy); recurrent (recurrence after prior line of therapy and complete/partial response with most recent prior therapy); and secondary refractory (recurrence after prior line of therapy and no partial/complete response with most recent prior therapy). Tumors progressing during maintenance therapy (e.g., bevacizumab or nintedanib) were not considered as refractory to treatment (excluding progression during endocrine therapy for endometrial cancer [EC]).

The number of prior lines of systemic therapy for advanced disease was defined as a dichotomous variable with two categories: 1 *vs* ≥2. Maintenance therapy (e.g., bevacizumab) was considered as part of the preceding line of therapy (i.e., not counted independently, excluding endocrine therapy for EC). Systemic therapy prior to the advanced or refractory setting was not included as a separate line of systemic therapy.

ECs were traditionally classified as type I or type II; type I comprised grades 1 and 2 endometrioid carcinomas, whereas type II included grade 3 endometrioid, serous, and clear cell carcinomas. However, grades 1 and 2 endometrioid carcinomas with abnormal p53 immunohistochemistry (IHC) expression pattern were also considered type II.

The histomolecular classification of ECs was performed as recently described (2, 3); patients were classified into one of these five distinct subgroups: deoxyribonucleic acid polymerase epsilon mutated (*POLE*mut), microsatellite instability-high (MSI-H), p53 abnormal (p53abn), non-specific molecular profile (NSMP), and histomolecular testing not done or inconclusive (NOS). Patients with multiple-classifier EC were allocated in one of these five subgroups, as described by León-Castillo and colleagues (4).

*Efficacy*

Death or evident clinical progression before an imaging scan was acquired was defined as progressive disease both per immune-related response criteria (irRC) (irPD) and Response Evaluation Criteria in Solid Tumors, version 1.1 (RECIST v1.1) (PD).

Disease control rate (per irRC [irDCR] or RECIST v1.1[DCR]) was defined as the proportion of patients with complete response (irCR or CR), partial response (irPR or PR), and stable disease (irSD and SD) at week 26.

*Safety*

A dose-limiting toxicity was defined as any of the following treatment related events: (i) any grade 3 or higher allergic, autoimmune or injection site reaction or (ii) any grade 4 hematologic or non-hematologic toxicity except fever. The dose-limiting toxicity observation window was 30 days after the last study treatment component was given. If two or more unmanageable DLTs were observed within the first six enrolled patients until 30 days after the third stereotactic body radiotherapy fraction, irrespective of the cohort, the culprit drug(s) were to be reduced in dose (i.e., safety run-in).

A serious treatment-emerging adverse event (TEAE) was defined as any adverse event, whether or not considered study treatment related, occurring at any dose or during any use of the protocol-specified treatment that: (i) resulted in death, except due to progression of the cancer under study; (ii) was life threatening; (iii) resulted in persistent or significant disability/incapacity; (iv) resulted in or prolonged an existing inpatient hospitalization; (v) was a congenital anomaly/birth defect; or (vi) was another important medical event.

The Data Safety Monitoring Board reviewed the safety data at least once yearly. Decisions to withhold or reduce dose of any treatment component were made as outlined in the protocol.

*Immunohistochemical and Molecular Analysis*

In the cervical cohort, tumors were interpreted as PD-L1 positive if the combined positive score (number of PD-L1–positive cells [tumor cells, macrophages, lymphocytes] divided by the total number of tumor cells, multiplied by 100) was 1 or greater.

In the endometrial cohort, tumors were interpreted as hormone receptor positive when immunoreactivity for estrogen receptor or progesterone receptors was found in more than 1% of carcinoma cells. The phosphatase and tensin homolog (PTEN) IHC pattern was interpreted as described by Wang, *et al*: absent, equivocal, reduced, retained, or subclonal loss (5). These findings were dichotomized for data analysis and visualization: absent (absent and subclonal loss) *vs* present (equivocal, reduced, and retained). Similarly, p53 IHC pattern was also dichotomized: abnormal (overexpression, complete absence, cytoplasmatic, and inconclusive [preference overexpression]) *vs* wild type (wild type and inconclusive [preference wild type]). MSI status and *POLE* mutational status were assessed using a targeted amplicon-based panel consisting of eight homopolymers stretches frequently mutated in MSI-H tumors and the *POLE* P286R and V411L mutations (6, 7). Amplicon-based enriched libraries were sequenced on an Illumina HiSeq4000 in a v3 Flow Cell yielding 2x151bp paired-end reads. We obtained on average 1,488,178 (standard deviation; 182,506) paired-end reads per sample. For each targeted region the wildtype and mutated reads were directly counted from the raw sequences to identify the mutations.

*Systemic Inflammatory Markers*

The derived neutrophil to lymphocyte ratio (dNLR) was calculated via the following equation: absolute neutrophil count/(white blood cell count − absolute neutrophil count). The cutoff for a poor dNLR was greater than 3 (according to the cutoff from the largest published study with immune checkpoint inhibitors in patients with cancer) (8, 9). The dNLR was deemed more relevant than NLR because it includes monocytes, eosinophils, and other leukocyte subpopulations.

The lung immune prognostic index (LIPI) was developed on the basis of dNLR greater than 3 and lactate dehydrogenase greater than the upper limit of normal, characterizing three groups (good, no factors; intermediate, one factor; poor, two factors) (10).

The C-reactive protein to albumin ratio (CAR) was calculated by dividing the serum C-reactive protein level by the serum albumin level (11). The optimal cutoff value for the C-reactive protein to albumin ratio (0.225) was calculated using receiver operating characteristic curve analysis (area under the curve, 0.651) on the basis of the Youden index (sensitivity 0.86; specificity, 0.65).

The modified Glasgow prognostic score (mGPS) was developed on the basis of an increased serum C-reactive protein level (>10mg/L) and hypoalbuminaemia (<35g/L), characterizing three groups (good, no factors; intermediate, one factor; poor, two factors) (12).

# Additional Statistical Analyses

*General*

The informational design of the figures includes measures to limit visual exaggeration of results (13, 14).

*Subgroup Analyses*

Efficacy analyses were also performed according to both prespecified and *post hoc* subgroups (online supplemental table S10) by the Fisher’s exact test, generalized logrank test of Sun, and logrank test.

*Health-Related Quality of Life*

For all (sub)scales and symptom indices, the higher the score the better the health-related quality of life. The minimal clinically important difference (MID; i.e., the difference perceived by patients as important, and by clinicians to require a change in the patients’ management) for Functional Assessment of Cancer Therapy-Cervix (FACT-Cx, version 4.0) and FACT-General (FACT-G, version 4.0) were chosen as described elsewhere (15). In short, the MID for the physical, social/familial, emotional, and functional subscales was 3 points, whereas it was 6 points for the cervical cancer subscale. The MID for the total score was 11 points for the cervical cohort (FACT-Cx total) and 7 points for the endometrial cohort (FACT-G total). If there were missing values, subscale scores were prorated when more than 50% of the items were answered; the total score was then calculated as the sum of the un-weighted subscale scores.

*Translational Work*

Differences between responders and nonresponders were performed using the Fisher’s exact test for categorical variables and the Mann-Whitney/Wilcoxon Rank-Sum test for continuous variables. Patients with an irPR or irCR per irRC at week 26 were considered as responders.

# Extended Full Analysis Set of the Cervical Cohort

*Patient Characteristics*

The patient (cervical, case ID 1) not included in the full analysis set was a 29-year-old female who had been initially diagnosed with International Federation of Gynecology and Obstetrics stage IB2 human papillomavirus-positive squamous cell carcinoma of the cervix (grade, unknown) for which she received concurrent chemoradiotherapy with brachytherapy. Two months after treatment completion, she had a symptomatic recurrence (disease status, primary refractory); two months thereafter, she was diagnosed with metastatic disease. She was subsequently enrolled in the PRIMMO study. She progressed (both per irRC and RECIST v1.1) 11 days after study enrollment; she died 12 days thereafter. She did not receive any pembrolizumab dose or stereotactic body radiotherapy fraction within the present study.

*Efficacy*

In the extended full analysis set (eFAS), week 26 responses per irRC were seen in 2 out of 19 (10.5%) (90% CI, 1.9 to 29.6) patients, whereas those per RECIST v1.1 were seen in 3 out of 19 (15.8%) (90% CI, 4.5 to 35.9) patients.

Median interval-censored progression-free survival per irRC (irPFS) in the eFAS was 1.9 weeks (95% CI, 1.9 to 25.7) with 26-week, 52-week, and 78-week interval-censored irPFS rates of 10.5% (95% CI, 2.6 to 34.0).

Median overall survival in the eFAS was 39.6 weeks (95% CI, 13.1 to 67.0) with 26-week, 52-week, and 78-week overall survival rates of 62.7% (95% CI, 37.3 to 80.2), 33.3% (95% CI, 13.2 to 55.0), and 22.2% (95% CI, 4.9 to 47.1), respectively.

*Safety*

The non-evaluable patient had two TEAEs (both were disease progression, the second one resulting in death; both were serious TEAEs) but none was treatment related.

*Health-Related Quality of Life*

Health-related quality of life data for the eFAS are reported in online supplemental tables S8 and S9 and online supplemental figure S6.

# Supplementary Figures

Figure S1. Immunomodulatory Drug Cocktail Compliance Rate over Time

Data are proportion±standard error.

IDC, immunomodulatory five-drug cocktail.

Figure S2. Right-Censored Progression-Free Survival per Immune-Related Response Criteria

Median right-censored irPFS was 13.4 (95% CI, 11.3 to 26.1) weeks in the cervical cohort and 13.1 (95% CI, 13.1 to 19.4) weeks in the endometrial cohort.

Figure S3. Kaplan-Meier Analysis of Overall Survival by Disease Cohort.

Figure S4. Efficacy per Subgroup (Cervical Cohort)

Interval plot showing (A) objective response rate per immune-related response criteria (irORR), (B) objective response rate per Response Evaluation Criteria in Solid Tumors, version 1.1 (ORR), (C) interval-censored progression-free survival per immune-related response criteria, and (D) overall survival in different subgroups. Data are number of patients in the subgroup followed by % (for response outcomes) or median (for time-to-event outcomes) with corresponding confidence intervals between brackets. Horizontal line represents the confidence interval. The vertical full reference line represents the primary objective (an irORR with a lower bound of the 90% confidence interval of >10%). The vertical dotted reference lines represent the efficacy in the overall cohort.

HPV, human papillomavirus; NE, not estimable; ORR, objective response rate; OS, overall survival; PD-L1, programmed death-ligand 1; PFS, progression-free survival; sTILs, stromal tumor-infiltrating lymphocytes.

Figure S5. Efficacy per Subgroup (Endometrial Cohort)

Interval plot showing (A) objective response rate per immune-related response criteria (irORR), (B) objective response rate per Response Evaluation Criteria in Solid Tumors, version 1.1, (C) interval-censored progression-free survival per immune-related response criteria, and (D) overall survival in different subgroups. Data are number of patients in the subgroup followed by % (for response outcomes) or median (for time-to-event outcomes) with corresponding confidence intervals between brackets. The vertical full reference line represents the primary objective (an irORR with a lower bound of the 90% confidence interval of >10%). The vertical dotted reference lines represent the efficacy in the overall cohort.

MS, microsatellite status; MSI, microsatellite instability; NE, not estimable; NOS, not otherwise specified; NSMP, no specific molecular profile; OS, overall survival; PFS, progression-free survival; PTEN, phosphatase and tensin homolog; sTILs, stromal tumor-infiltrating lymphocytes.

Fig S6. Quality-of-Life Scores (Total and for Each Subscale) by Disease Cohort

(A-F) Data are mean±standard deviation for the intention-to-treat analysis sets. (F) Total score refers to the FACT-Cx total score (range, 0 to 168) for the cervical cohort and FACT-G total score (range, 0 to 108) for the endometrial cohort.

Fig S7. Differences in Systemic Inflammatory Markers Between Responders and Nonresponders

Data on top of the bars are number of patients (%). Patients were grouped according to their week 26 response per immune-related response criteria (irRC). The Fisher’s exact p-values (comparing responders to nonresponders) are (A) p=.320, (B) p=.572, (C) p=.150, and (D) p=.573, respectively.

CAR, C-reactive protein to albumin ratio; CR, complete response; dLNR, derived lymphocyte to neutrophil ratio; irRC, immune-related response criteria; LIPI, lung immune prognostic index; mGPS, modified Glasgow prognostic score; PD, progressive disease; PR, partial response; SD, stable disease.

# Supplementary Tables

Table S1. Summary of Studies with Single-Agent Immune Checkpoint Inhibitor According to Biomarker Status in Cervical and Endometrial Cancer

| **Study name or identifier** | **Patient population (No. of prior lines)** | **Single-agent ICI** | **n** | **ORR** | **mDOR** | **mPFS** | **mOS** |
| --- | --- | --- | --- | --- | --- | --- | --- |
| Cervical |  |  |  |  |  |  |  |
| PD-L1^+^ |  |  |  |  |  |  |  |
| KEYNOTE-028 (16) | Advanced (≥1L) | Pembrolizumab | 24 | 17% | 5.4 mo | 2 mo | 11 mo |
| KEYNOTE-158 (17) | Advanced (≥1L) | Pembrolizumab | 82 | 14% | NR | 2.1 mo | 11 mo |
| NRG-GY002 (18) | Persistent/recurrent/metastatic (≥1L) | Nivolumab | 5 | (0%) | – | – | – |
| CHECKMATE-358 (19) | Recurrent/  metastatic HPV^+^  (1-3L) | Nivolumab | 10 | 20% | NR | – | – |
| NCT03104699 (20) | Recurrent/  metastatic (≥1L) | Balstilimab | 99 | 20% | – | – | – |
| JapicCTI-163212 (21) | Advanced/recurrent | Nivolumab | 15 | 33% | – | – | – |
| EMPOWER (22) | Recurrent/  Metastatic (2L) | Cemiplimab | 82 | 18% | – | 3.0 mo | 13.9 mo |
| PD-L1^-^ |  |  |  |  |  |  |  |
| KEYNOTE-158 (17) | Advanced (≥1L) | Pembrolizumab | 15 | 0% | – | – | – |
| NRG-GY002 (18) | Persistent/recurrent/metastatic (≥1L) | Nivolumab | 17 | 6% | – | – | – |
| CHECKMATE-358 (19) | Recurrent/  metastatic HPV^+^  (1-3L) | Nivolumab | 6 | 17% | NR | – | – |
| NCT03104699 (20) | Recurrent/  Metastatic (≥1L) | Balstilimab | 43 | 8% | – | – | – |
| JapicCTI-163212 (21) | Advanced/recurrent | Nivolumab | 5 | (0%) | – | – | – |
| EMPOWER (22) | Recurrent/  Metastatic (2L) | Cemiplimab | 44 | 11% | – | 1.9 mo | 7.7 mo |
| Unselected/unknown |  |  |  |  |  |  |  |
| Rischin, *et al.* (23) | Recurrent/  metastatic | Cemiplimab | 10 | 10% | 11.2 mo | 1.9 mo | 10.3mo |
| CHECKMATE-358 (19) | Recurrent/  metastatic HPV^+^  (1-3L) | Nivolumab | 3 | (67%) | (NR) | – | – |
| NCT01693783 (24) | Recurrent/  metastatic (≥1L) | Ipilimumab | 42 | 9% | – | 2.5 mo | 8.5 mo |
| Endometrial |  |  |  |  |  |  |  |
| dMMR/MSI-H |  |  |  |  |  |  |  |
| KEYNOTE-158 (25) | Advanced (≥1L) | Pembrolizumab | 49 | 57% | NR | 26 mo | NR |
| PHAEDRA (26) | Advanced/recurrent (≤3L) | Durvalumab | 36 | 47% | – | 8.3 mo | NR |
| GARNET (27) | Advanced/recurrent diagnosis (≥1L) | Dostarlimab | 108 | 47% | NR | – | – |
| NCT02912572 (28) | Recurrent (≥1L) | Avelumab | 14 | 27% | – | 6-mo: 40% | NR |
| pMMR/MSS |  |  |  |  |  |  |  |
| PHAEDRA (26) | Advanced/recurrent (1-3L) | Durvalumab | 35 | 3% | – | 1.8 mo | 12 mo |
| GARNET (27) | Advanced/recurrent (≥1L) | Dostarlimab | 142 | 19% | – | – | – |
| NCT02912572 (28) | Recurrent (≥1L) | Avelumab | 16 | 6% | – | 6-mo: 6% | 7 mo |
| NCI10104 (29) | Advanced/recurrent/metastatic (≥2L) | Nivolumab | 18 | 11% | – | 1.9 mo | 7.9 mo |
| Unselected/unknown |  |  |  |  |  |  |  |
| KEYNOTE-028 (30) | Locally advanced/metastatic PD-L1^+^ | Pembrolizumab | 24 | 13% | NR | 1.8 mo | NR |
| NCT01375842 (31) | Locally advanced/recurrent/metastatic | Atezolizumab | 15 | 13% | NR | 1.7 mo | 9.6 mo |
| JapicCTI-163212 (21) | Advanced/recurrent | Nivolumab | 23 | 23% | – | 3.4 mo | 6-mo:  73% |

PD-L1, programmed cell death receptor-ligand 1; HPV, human papillomavirus; ICI, immune checkpoint inhibitor; mOS, median overall survival; mPFS, median progression-free survival; NR: not reached; ORR, objective response rate.

Table S2. Immunological Rationale for the Immunomodulatory Five-Drug Cocktail

| **IDC component** | **Mechanism** | **Most advanced study setting(s)** |
| --- | --- | --- |
| Low- dose cyclophosphamide (reviewed in (32, 33)) |  |  |
|  | Induction of immunogenic cell death | *In vivo* and human (34) |
|  | Potentiation of dendritic cell function | *In vivo* (35) |
|  | Polarization of T cells to T helper 1 and/or T helper 17 phenotype | *In vivo* (36, 37) |
|  | Decrease of regulatory T cell/effector T cell ratio | *In vivo* and human (36, 38-42) |
|  | Production of type I IFN and enhances number of memory T cells | *In vivo* (43, 44) |
|  | Induction of innate immunity-induced tumor regression | *In vivo* (45-47) |
|  | Induction of tumor-specific T-cell responses | *In vivo* and human (40, 44, 48) |
|  | Tumor vasculature remodeling | *In vivo* (49, 50) |
|  | Synergism with cancer vaccines | *In vivo* and human (51-56) |
|  | Synergism with antibodies against co-stimulatory molecules | *In vivo* (57, 58) |
|  | Synergism with anti–PD-1 | *In vivo* and human (48, 59, 60) |
| Aspirin  (reviewed in (61)) |  |  |
|  | Reduction of PGE_2_ synthesis | *In vivo* and human (62, 63) |
|  | Reduction of PD-L1 expression on cancer cells | *In vitro* (64) |
|  | Reduction of VEGF levels and/or tumor angiogenesis | *In vivo* and/or human (65-67) |
|  | Synergism with anti–PD-1 | *In vivo* and human (60, 62, 68) |
|  | Reduction of platelet-mediated shielding of circulating cancer cells | *In vitro* and human (69, 70) |

| Lansoprazole (reviewed in (71, 72)) |  |  |
| --- | --- | --- |
|  | Buffering tumor acidosis to restore innate and adaptive antitumor immunity | *In vivo* (73-75) |
| Vitamin D  (reviewed in (76)) |  |  |
|  | Reduction of tumor angiogenesis | *In vivo* (77-79) |
|  | Stromal remodeling and/or reprogramming | *In vivo* (80-82) |
|  | Potentiation of NK-cell function | *In vitro* (83) |
|  | Increase in immune cell infiltration and reduction of suppressive CD34^+^ cells | Human (84, 85) |
|  | Synergism with a tumor vaccine (and low-dose cyclophosphamide) | *In viv*o (86) |
|  | Upregulation of genes involved in immune response, inflammation, and cell adhesion | Human (87), (88, 89) |
| Curcumin (reviewed in (90, 91)) |  |  |
|  | Radiosensitizer | *In vivo* (92) |
|  | Reduction of regulatory T cells | *In vitro* (93) |
|  | Reduction of VEGF levels and/or tumor angiogenesis | *In vitro* and/or *in vivo* (94, 95) |
|  | Enhance antitumor immunity (mainly NF-κB–mediated) | *In vitro* and *in vivo* (96-99) |
|  | Inhibition of inflammation-mediated PD-L1 expression | *In vivo* (99) |
|  | Synergism with anti–PD-1/PD-L1 | *In vivo* (100) |

Note that this list is nonexhaustive.

CD, cluster of differentiation; IDC, immunomodulatory five-drug cocktail; IFN, interferon; NF-κB, nuclear factor kappa-light-chain-enhancer of activated B cells; NK, natural killer; PD-1, programmed death receptor-1; PD-L1, programmed death ligand-1; PGE, prostaglandin E_2_, VEGF, vascular endothelial growth factor

Table S3. List and Details of Primary Antibodies for Immunohistochemistry

| **Antibody target** | **Antibody name/catalog or lot number** | **Supplier** | **Type of antibody/concentration as reported by manufacturer** |
| --- | --- | --- | --- |
| p16 | E6H4/#G32346 | Roche | Mouse monoclonal IgG/RTU |
| PD-L1 | E1L3N/#13684 | Cell Signaling Technology | Rabbit monoclonal IgG/  1:100 to 1:400 |
| ER | SP1/#H03685 | Roche | Rabbit monoclonal IgG/RTU |
| PgR | 1E2/#G26491 | Roche | Rabbit monoclonal IgG/RTU |
| PTEN | D4.3/#6 | Cell Signaling Technology | Rabbit monoclonal IgG/1:50 |
| p53 | DO7/#H10832 | Roche | Mouse monoclonal IgG/RTU |

ER, estrogen receptor; PD-L1, programmed death ligand-1; PgR, progesterone receptor; PTEN, phosphatase and tensin homolog; RTU, ready to use.

Table S4. List and Details of Fluorescently Conjugated Anti-Human Monoclonal Antibodies

| **Antibody target** | **Fluorochrome** | **Clone** | **Isotype/light chain** | **Supplier** |
| --- | --- | --- | --- | --- |
| Fixable viability dye | eFluor506 | – | – | Thermo Fisher |
| CD4 | Fluorescein isothiocyanate | RPA-T4 | Mouse IgG1/κ | Biolegend |
| CD127 | PerCP-Cy5.5 | HIL-7R-M21 | Mouse IgG1/κ | BD Biosciences |
| CD3 | PE/Cy7 | HIT3a | Mouse IgG2a/κ | Biolegend |
| CD8 | APC-H7 | SK1 | Mouse IgG1/κ | BD Biosciences |
| CD45 | Pacific Blue | HI30 | Mouse IgG1/κ | Biolegend |
| CD25 | PE | 2A3 | Mouse IgG1/κ | BD Biosciences |
| FoxP3 | APC | 236A/E7 | Mouse IgG1/κ | Thermo Fisher |

APC, allophycocyanin; CD, cluster of differentiation.

Table S5. Accrual Number per Participating Center, Overall and by Disease Cohort

| **ID including center identifier** | **Cervical**  **(n=18)** | **Endometrial (n=25)** | **Overall**  **(n=43)** |
| --- | --- | --- | --- |
| A | 9 | 11 | 20 |
| B | 2 | 5 | 7 |
| C | 2 | 4 | 6 |
| D | 5 | 5 | 10 |

Data are number of patients.

Table S6. SBRT Quality and Compliance by Disease Cohort.

|  | **Cervical (n=18)** | **Endometrial (n=25)** |
| --- | --- | --- |
| Dose received by 90% of the GTV (Gy), median (IQR) | 25.2 (24.8 to 26.1) | 26.5 (25.6 to 27.0) |
| Dose received by 90% of the PTV (Gy), median (IQR) | 24.0 (24.0 to 24.0) | 24.0 (24.0 to 24.2) |
| All dose >31.5Gy within PTV | 18 (100.0) | 25 (100.0) |
| All dose >12Gy within PTV + 3cm margin | 16 (88.9) | 22 (88.0) |
| SBRT interrupted | 1 (5.6) | 0 |
| Toxicity | 0 | 0 |
| Patient refusal | 0 | 0 |
| Administrative | 0 | 0 |
| Technical | 0 | 0 |
| Other | 1 (5.6) | 0 |
| SBRT stopped | 0 | 0 |

Data are number of patients (%), unless otherwise indicated.

GTV, gross tumor volume; IQR, interquartile range; PTV, planning target volume; SBRT, stereotactic body radiotherapy.

Table S7. Study Continuation Upon Progression

| **Progression** | **Cervical (n=19)** | **Endometrial (n=25)** |
| --- | --- | --- |
| Per irRC |  |  |
| No irPD | 2 | 2 |
| irPD | 17 | 23 |
| Discontinued study at initial progression | 14 | 16 |
| Discontinued study at first confirmation of progression | 3 | 6 |
| Discontinued study at second or later confirmation of progression | 0 | 1 |
| Per RECIST v1.1 |  |  |
| No PD | 2 | 2 |
| PD | 17 | 23 |
| Discontinued study at initial progression | 13 | 16 |
| Discontinued study at first confirmation of progression | 4 | 6 |
| Discontinued study at second or later confirmation of progression | 0 | 1 |

Data are number of patients.

irRC, immune-related response criteria; PD, progressive disease; RECIST v1.1, Response Evaluation Criteria in Solid Tumors, version 1.1.

Table S8. Completion rates of Quality-of-Life Questionnaires by Disease Cohort

| **Time point** | **Cervical** | **Endometrial** |
| --- | --- | --- |
| Baseline | 19/19 (100.0) | 24/25 (96.0) |
| Week 12 | 12/14 (85.7) | 12/18 (66.7) |
| Week 26 | 4/5 (80.0) | 4/6 (66.7) |
| Week 38 | 2/5 (40.0) | 2/5 (40.0) |

Data are number of patients (%). Patients were “expected” to complete questionnaires until they discontinued the study.

Table S9. Categorized Quality-of-Life Scores (Total and for Each Subscale) by Disease Cohort

| **HRQOL** | **Week 12** | | **Week 26** | | **Week 38** | |
| --- | --- | --- | --- | --- | --- | --- |
|  | **Cervical** | **Endo-metrial** | **Cervical** | **Endo-metrial** | **Cervical** | **Endo-metrial** |
| Total |  |  |  |  |  |  |
| Decrease | 4 (33.3) | 3 (27.3) | 1 (25.0) | 1 (25.0) | 0 | 0 |
| Stable | 7 (58.3) | 7 (63.6) | 3 (75.0) | 2 (50.0) | 1 (100.0) | 1 (100.0) |
| Increase | 1 (8.3) | 1 (9.1) | 0 | 1 (25.0) | 0 | 0 |
| Subscale |  |  |  |  |  |  |
| Physical |  |  |  |  |  |  |
| Decrease | 3 (25.0) | 5 (41.7) | 2 (50.0) | 1 (25.0) | 1 (50.0) | 0 |
| Stable | 8 (66.7) | 4 (33.3) | 2 (50.0) | 1 (25.0) | 1 (50.0) | 1 (50.0) |
| Increase | 1 (8.3) | 3 (25.0) | 0 (0) | 2 (50.0) | 0 | 1 (50.0) |
| Social/familial |  |  |  |  |  |  |
| Decrease | 3 (25.0) | 1 (9.1) | 0 | 0 | 0 | 0 |
| Stable | 9 (75.0) | 8 (72.7) | 4 (100.0) | 4 (100.0) | 2 (100.0) | 1 (100.0) |
| Increase | 0 | 2 (18.2) | 0 | 0 | 0 | 0 |
| Emotional |  |  |  |  |  |  |
| Decrease | 2 (16.7) | 1 (8.3) | 2 (50.0) | 0 | 1 (50.0) | 0 |
| Stable | 7 (58.3) | 6 (50.0) | 1 (25.0) | 3 (75.0) | 1 (50.0) | 1 (50.0) |
| Increase | 3 (25.0) | 5 (41.7) | 1 (250) | 1 (25.0) | 0 | 1 (50.0) |
| Functional |  |  |  |  |  |  |
| Decrease | 5 (41.7) | 6 (50.0) | 2 (50.0) | 3 (75.0) | 1 (50.0) | 2 (100.0) |
| Stable | 6 (50.0) | 5 (41.7) | 2 (50.0) | 0 | 1 (50.0) | 0 |
| Increase | 1 (8.3) | 1 (8.3) | 0 | 1 (25.0) | 0 | 0 |
| Cervical cancer |  |  |  |  |  |  |
| Decrease | 4 (33.3) | – | 0 | – | 0 | – |
| Stable | 8 (66.7) | – | 4 (100.0) | – | 1 (100.0) | – |
| Increase | 0 | – | 0 | – | 0 | – |

Table S10. Prespecified and *Post Hoc* Subgroups By Disease Cohort

|  | **Cervical** | **Endometrial** |
| --- | --- | --- |
| **Prespecified** | Histology  Squamous *vs* non-squamous | Histology  Endometrioid *vs* non-endometrioid |
|  | PD-L1 status  Positive *vs* negative | Grade  Low grade *vs* high grade |
|  | HPV status  Positive *vs* negative | Hormone receptor status  Positive *vs* negative |
|  |  | PTEN status  Present *vs* absent |
|  |  | p53 status  Abnormal *vs* wildtype |
|  |  | Microsatellite instability status  MSI *vs* MSS |
|  |  | *POLE* status  *POLE*mut *vs* *POLE*wt |
| ***Post hoc*** | Prior lines  1 *vs* ≥2 | Prior lines  1 *vs* ≥2 |
|  | Disease status  Primary refractory *vs* recurrent *vs* secondary refractory | Disease status  Primary refractory *vs* recurrent *vs* secondary refractory |
|  | sTILs  0-10% *vs* 20-40% *vs* 50-90% | sTILs  0-10% *vs* 20-40% *vs* 50-90% |
|  |  | Traditional classification  Type I *vs* type II |
|  |  | Histomolecular classification  *POLE*mut *vs* MSI *vs* p53abn *vs* NSMP *vs* NOS |

HPV, human papillomavirus; MSI, microsatellite instability; MSS, microsatellite stable; NOS, not otherwise specified; NSMP, no specific molecular profile; PD-L1, programmed death ligand-1; *POLE*, deoxyribonucleic acid polymerase epsilon; PTEN, phosphatase and tensin homolog; sTILs, stromal tumor-infiltrating lymphocytes.

# References

1. Benedict SH, Yenice KM, Followill D, Galvin JM, Hinson W, Kavanagh B, et al. Stereotactic body radiation therapy: the report of AAPM Task Group 101. Med Phys. 2010;37(8):4078-101.

2. León-Castillo A, de Boer SM, Powell ME, Mileshkin LR, Mackay HJ, Leary A, et al. Molecular Classification of the PORTEC-3 Trial for High-Risk Endometrial Cancer: Impact on Prognosis and Benefit From Adjuvant Therapy. Journal of Clinical Oncology. 2020;38(29):3388-97.

3. Vermij L, Smit V, Nout R, Bosse T. Incorporation of molecular characteristics into endometrial cancer management. Histopathology. 2020;76(1):52-63.

4. León-Castillo A, Gilvazquez E, Nout R, Smit VT, McAlpine JN, McConechy M, et al. Clinicopathological and molecular characterisation of 'multiple-classifier' endometrial carcinomas. The Journal of pathology. 2020;250(3):312-22.

5. Wang L, Piskorz A, Bosse T, Jimenez-Linan M, Rous B, Gilks CB, et al. Immunohistochemistry and Next-generation Sequencing Are Complementary Tests in Identifying PTEN Abnormality in Endometrial Carcinoma Biopsies. Int J Gynecol Pathol. 2021.

6. De Craene B, Van de Velde J, Bellon E, Gazin M, Rondelez E, Vandenbroeck L, et al. Detection of microsatellite instability (MSI) with a novel set of 7 Idylla biomarkers on colorectal cancer samples in a multi-center study. Annals of Oncology. 2018;29:viii51.

7. Zhao H, Thienpont B, Yesilyurt BT, Moisse M, Reumers J, Coenegrachts L, et al. Mismatch repair deficiency endows tumors with a unique mutation signature and sensitivity to DNA double-strand breaks. Elife. 2014;3:e02725.

8. Valero C, Lee M, Hoen D, Weiss K, Kelly DW, Adusumilli PS, et al. Pretreatment neutrophil-to-lymphocyte ratio and mutational burden as biomarkers of tumor response to immune checkpoint inhibitors. Nature Communications. 2021;12(1):729.

9. Templeton AJ, McNamara MG, Šeruga B, Vera-Badillo FE, Aneja P, Ocaña A, et al. Prognostic role of neutrophil-to-lymphocyte ratio in solid tumors: a systematic review and meta-analysis. Journal of the National Cancer Institute. 2014;106(6):dju124.

10. Mezquita L, Auclin E, Ferrara R, Charrier M, Remon J, Planchard D, et al. Association of the Lung Immune Prognostic Index With Immune Checkpoint Inhibitor Outcomes in Patients With Advanced Non–Small Cell Lung Cancer. JAMA Oncol. 2018;4(3):351-7.

11. Fairclough E, Cairns E, Hamilton J, Kelly C. Evaluation of a modified early warning system for acute medical admissions and comparison with C-reactive protein/albumin ratio as a predictor of patient outcome. Clin Med (Lond). 2009;9(1):30-3.

12. McMillan DC. The systemic inflammation-based Glasgow Prognostic Score: a decade of experience in patients with cancer. Cancer treatment reviews. 2013;39(5):534-40.

13. Castanon Alvarez E, Aspeslagh S, Soria JC. 3D waterfall plots: a better graphical representation of tumor response in oncology. Annals of Oncology. 2017;28(3):454-6.

14. Kim MS, Prasad V. Assessment of Accuracy of Waterfall Plot Representations of Response Rates in Cancer Treatment Published in Medical Journals. JAMA Netw Open. 2019;2(5):e193981-e.

15. Yost KJ, Eton DT. Combining distribution- and anchor-based approaches to determine minimally important differences: the FACIT experience. Eval Health Prof. 2005;28(2):172-91.

16. Frenel JS, Le Tourneau C, O'Neil B, Ott PA, Piha-Paul SA, Gomez-Roca C, et al. Safety and Efficacy of Pembrolizumab in Advanced, Programmed Death Ligand 1-Positive Cervical Cancer: Results From the Phase Ib KEYNOTE-028 Trial. Journal of Clinical Oncology. 2017;35(36):4035-41.

17. Chung HC, Ros W, Delord J-P, Perets R, Italiano A, Shapira-Frommer R, et al. Efficacy and Safety of Pembrolizumab in Previously Treated Advanced Cervical Cancer: Results From the Phase II KEYNOTE-158 Study. Journal of Clinical Oncology. 2019;37(17):1470-8.

18. Santin AD, Deng W, Frumovitz M, Buza N, Bellone S, Huh W, et al. Phase II evaluation of nivolumab in the treatment of persistent or recurrent cervical cancer (NCT02257528/NRG-GY002). Gynecologic Oncology. 2020;157(1):161-6.

19. Naumann RW, Hollebecque A, Meyer T, Devlin M-J, Oaknin A, Kerger J, et al. Safety and Efficacy of Nivolumab Monotherapy in Recurrent or Metastatic Cervical, Vaginal, or Vulvar Carcinoma: Results From the Phase I/II CheckMate 358 Trial. Journal of Clinical Oncology. 2019;37(31):2825-34.

20. O'Malley DM, Oaknin A, Monk BJ, Selle F, Rojas C, Gladieff L, et al. Phase II study of the safety and efficacy of the anti-PD-1 antibody balstilimab in patients with recurrent and/or metastatic cervical cancer. Gynecologic Oncology. 2021.

21. Hasegawa K, Tamura K, Katsumata N, Matsumoto K, Takahashi S, Mukai H, et al. Efficacy and safety of nivolumab (Nivo) in patients (pts) with advanced or recurrent uterine cervical or corpus cancers. Journal of Clinical Oncology. 2018;36(15_suppl):5594-.

22. Tewari KS, Monk BJ, Vergote I, Miller A, de Melo AC, Kim H-S, et al. Survival with Cemiplimab in Recurrent Cervical Cancer. New England Journal of Medicine. 2022;386(6):544-55.

23. Rischin D, Gil-Martin M, González-Martin A, Braña I, Hou JY, Cho D, et al. PD-1 blockade in recurrent or metastatic cervical cancer: Data from cemiplimab phase I expansion cohorts and characterization of PD-L1 expression in cervical cancer. Gynecologic Oncology. 2020;159(2):322-8.

24. Lheureux S, Butler MO, Clarke B, Cristea MC, Martin LP, Tonkin K, et al. Association of Ipilimumab With Safety and Antitumor Activity in Women With Metastatic or Recurrent Human Papillomavirus-Related Cervical Carcinoma. JAMA Oncol. 2018;4(7):e173776.

25. Marabelle A, Le DT, Ascierto PA, Giacomo AMD, Jesus-Acosta AD, Delord J-P, et al. Efficacy of Pembrolizumab in Patients With Noncolorectal High Microsatellite Instability/Mismatch Repair–Deficient Cancer: Results From the Phase II KEYNOTE-158 Study. Journal of Clinical Oncology. 2020;38(1):1-10.

26. Antill Y, Kok P-S, Robledo K, Yip S, Cummins M, Smith D, et al. Clinical activity of durvalumab for patients with advanced mismatch repair-deficient and repair-proficient endometrial cancer. A nonrandomized phase 2 clinical trial. J Immunother Cancer. 2021;9(6):e002255.

27. Oaknin A, Tinker AV, Gilbert L, Samouëlian V, Mathews C, Brown J, et al. Clinical Activity and Safety of the Anti-Programmed Death 1 Monoclonal Antibody Dostarlimab for Patients With Recurrent or Advanced Mismatch Repair-Deficient Endometrial Cancer: A Nonrandomized Phase 1 Clinical Trial. JAMA Oncol. 2020;6(11):1766-72.

28. Konstantinopoulos PA, Luo W, Liu JF, Gulhan DC, Krasner C, Ishizuka JJ, et al. Phase II Study of Avelumab in Patients With Mismatch Repair Deficient and Mismatch Repair Proficient Recurrent/Persistent Endometrial Cancer. Journal of Clinical Oncology. 2019;37(30):2786-94.

29. Lheureux S, Matei D, Konstantinopoulos PA, Block MS, Jewell A, Gaillard S, et al. A randomized phase II study of cabozantinib and nivolumab versus nivolumab in recurrent endometrial cancer. Journal of Clinical Oncology. 2020;38(15_suppl):6010-.

30. Ott PA, Bang Y-J, Berton-Rigaud D, Elez E, Pishvaian MJ, Rugo HS, et al. Safety and Antitumor Activity of Pembrolizumab in Advanced Programmed Death Ligand 1–Positive Endometrial Cancer: Results From the KEYNOTE-028 Study. Journal of Clinical Oncology. 2017;35(22):2535-41.

31. Fleming GF, Emens LA, Eder JP, Hamilton EP, Liu JF, Liu B, et al. Clinical activity, safety and biomarker results from a phase Ia study of atezolizumab (atezo) in advanced/recurrent endometrial cancer (rEC). Journal of Clinical Oncology. 2017;35(15_suppl):5585-.

32. Madondo MT, Quinn M, Plebanski M. Low dose cyclophosphamide: Mechanisms of T cell modulation. Cancer Treatment Reviews. 2016;42:3-9.

33. Hughes E, Scurr M, Campbell E, Jones E, Godkin A, Gallimore A. T-cell modulation by cyclophosphamide for tumour therapy. Immunology. 2018;154(1):62-8.

34. Pol J, Vacchelli E, Aranda F, Castoldi F, Eggermont A, Cremer I, et al. Trial Watch: Immunogenic cell death inducers for anticancer chemotherapy. Oncoimmunology. 2015;4(4):e1008866.

35. Ding ZC, Blazar BR, Mellor AL, Munn DH, Zhou G. Chemotherapy rescues tumor-driven aberrant CD4+ T-cell differentiation and restores an activated polyfunctional helper phenotype. Blood. 2010;115(12):2397-406.

36. Matar P, Rozados VR, Gervasoni SI, Scharovsky GO. Th2/Th1 switch induced by a single low dose of cyclophosphamide in a rat metastatic lymphoma model. Cancer Immunology, Immunotherapy. 2002;50(11):588-96.

37. Viaud S, Flament C, Zoubir M, Pautier P, LeCesne A, Ribrag V, et al. Cyclophosphamide Induces Differentiation of Th17 Cells in Cancer Patients. Cancer Research. 2011;71(3):661-5.

38. Berd D, Mastrangelo MJ. Effect of low dose cyclophosphamide on the immune system of cancer patients: depletion of CD4+, 2H4+ suppressor-inducer T-cells. Cancer Research. 1988;48(6):1671-5.

39. North RJ. Cyclophosphamide-facilitated adoptive immunotherapy of an established tumor depends on elimination of tumor-induced suppressor T cells. J Exp Med. 1982;155(4):1063-74.

40. Scurr M, Pembroke T, Bloom A, Roberts D, Thomson A, Smart K, et al. Low-Dose Cyclophosphamide Induces Antitumor T-Cell Responses, which Associate with Survival in Metastatic Colorectal Cancer. Clinical Cancer Research. 2017;23(22):6771-80.

41. Zhao J, Cao Y, Lei Z, Yang Z, Zhang B, Huang B. Selective Depletion of CD4<sup>+</sup>CD25<sup>+</sup>Foxp3<sup>+</sup> Regulatory T Cells by Low-Dose Cyclophosphamide Is Explained by Reduced Intracellular ATP Levels. Cancer Research. 2010;70(12):4850-8.

42. van der Most RG, Currie AJ, Mahendran S, Prosser A, Darabi A, Robinson BWS, et al. Tumor eradication after cyclophosphamide depends on concurrent depletion of regulatory T cells: a role for cycling TNFR2-expressing effector-suppressor T cells in limiting effective chemotherapy. Cancer Immunology, Immunotherapy. 2008;58(8):1219.

43. Schiavoni G, Mattei F, Di Pucchio T, Santini SM, Bracci L, Belardelli F, et al. Cyclophosphamide induces type I interferon and augments the number of CD44hi T lymphocytes in mice: implications for strategies of chemoimmunotherapy of cancer. Blood. 2000;95(6):2024-30.

44. Wu J, Waxman DJ. Metronomic cyclophosphamide eradicates large implanted GL261 gliomas by activating antitumor Cd8(+) T-cell responses and immune memory. Oncoimmunology. 2015;4(4):e1005521.

45. Doloff JC, Waxman DJ. VEGF receptor inhibitors block the ability of metronomically dosed cyclophosphamide to activate innate immunity-induced tumor regression. Cancer Research. 2012;72(5):1103-15.

46. Ghiringhelli F, Ménard C, Terme M, Flament C, Taieb J, Chaput N, et al. CD4+CD25+ regulatory T cells inhibit natural killer cell functions in a transforming growth factor-beta-dependent manner. J Exp Med. 2005;202(8):1075-85.

47. Ghiringhelli F, Menard C, Puig PE, Ladoire S, Roux S, Martin F, et al. Metronomic cyclophosphamide regimen selectively depletes CD4+CD25+ regulatory T cells and restores T and NK effector functions in end stage cancer patients. Cancer Immunol Immunother. 2007;56(5):641-8.

48. Mkrtichyan M, Najjar YG, Raulfs EC, Abdalla MY, Samara R, Rotem-Yehudar R, et al. Anti-PD-1 synergizes with cyclophosphamide to induce potent anti-tumor vaccine effects through novel mechanisms. Eur J Immunol. 2011;41(10):2977-86.

49. Hamano Y, Sugimoto H, Soubasakos MA, Kieran M, Olsen BR, Lawler J, et al. Thrombospondin-1 associated with tumor microenvironment contributes to low-dose cyclophosphamide-mediated endothelial cell apoptosis and tumor growth suppression. Cancer Research. 2004;64(5):1570-4.

50. Mpekris F, Baish JW, Stylianopoulos T, Jain RK. Role of vascular normalization in benefit from metronomic chemotherapy. Proceedings of the National Academy of Sciences. 2017;114(8):1994-9.

51. MacLean GD, Miles DW, Rubens RD, Reddish MA, Longenecker BM. Enhancing the effect of THERATOPE STn-KLH cancer vaccine in patients with metastatic breast cancer by pretreatment with low-dose intravenous cyclophosphamide. J Immunother Emphasis Tumor Immunol. 1996;19(4):309-16.

52. Berd D, Maguire HC, Jr., Mastrangelo MJ. Induction of cell-mediated immunity to autologous melanoma cells and regression of metastases after treatment with a melanoma cell vaccine preceded by cyclophosphamide. Cancer Research. 1986;46(5):2572-7.

53. Denies S, Cicchelero L, Van Audenhove I, Sanders NN. Combination of interleukin-12 gene therapy, metronomic cyclophosphamide and DNA cancer vaccination directs all arms of the immune system towards tumor eradication. J Control Release. 2014;187:175-82.

54. Manrique SZ, Dominguez AL, Mirza N, Spencer CD, Bradley JM, Finke JH, et al. Definitive activation of endogenous antitumor immunity by repetitive cycles of cyclophosphamide with interspersed Toll-like receptor agonists. Oncotarget. 2016;7(28):42919-42.

55. Son CH, Shin DY, Kim SD, Park HS, Jung MH, Bae JH, et al. Improvement of antitumor effect of intratumoral injection of immature dendritic cells into irradiated tumor by cyclophosphamide in mouse colon cancer model. J Immunother. 2012;35(8):607-14.

56. Son CH, Bae JH, Lee HR, Shin DY, Yang K, Park YS. Enhanced dendritic cell-based immunotherapy using low-dose cyclophosphamide and CD25-targeted antibody for transplanted Lewis lung carcinoma cells. J Immunother. 2015;38(3):107-15.

57. Kim YH, Choi BK, Oh HS, Kang WJ, Mittler RS, Kwon BS. Mechanisms involved in synergistic anticancer effects of anti-4-1BB and cyclophosphamide therapy. Molecular Cancer Therapeutics. 2009;8(2):469-78.

58. Hirschhorn-Cymerman D, Rizzuto GA, Merghoub T, Cohen AD, Avogadri F, Lesokhin AM, et al. OX40 engagement and chemotherapy combination provides potent antitumor immunity with concomitant regulatory T cell apoptosis. J Exp Med. 2009;206(5):1103-16.

59. Zsiros E, Lynam S, Attwood KM, Wang C, Chilakapati S, Gomez EC, et al. Efficacy and Safety of Pembrolizumab in Combination With Bevacizumab and Oral Metronomic Cyclophosphamide in the Treatment of Recurrent Ovarian Cancer: A Phase 2 Nonrandomized Clinical Trial. JAMA Oncol. 2021;7(1):78-85.

60. Herrera FG, Ronet C, Ochoa de Olza M, Barras D, Crespo I, Andreatta M, et al. Low Dose Radiotherapy Reverses Tumor Immune Desertification and Resistance to Immunotherapy. Cancer Discovery. 2021.

61. Hamada T, Giannakis M, Ogino S. Aspirin in the era of immunotherapy. Oncotarget. 2017;8(43):73370-1.

62. Zelenay S, van der Veen AG, Böttcher JP, Snelgrove KJ, Rogers N, Acton SE, et al. Cyclooxygenase-Dependent Tumor Growth through Evasion of Immunity. Cell. 2015;162(6):1257-70.

63. Boutaud O, Sosa IR, Amin T, Oram D, Adler D, Hwang HS, et al. Inhibition of the Biosynthesis of Prostaglandin E2 By Low-Dose Aspirin: Implications for Adenocarcinoma Metastasis. Cancer Prevention Research (Philadelphia, Pa). 2016;9(11):855-65.

64. Zhang Y, Lv C, Dong Y, Yang Q. Aspirin-targeted PD-L1 in lung cancer growth inhibition. Thorac Cancer. 2020;11(6):1587-93.

65. Holmes CE, Jasielec J, Levis JE, Skelly J, Muss HB. Initiation of aspirin therapy modulates angiogenic protein levels in women with breast cancer receiving tamoxifen therapy. Clin Transl Sci. 2013;6(5):386-90.

66. Dai X, Yan J, Fu X, Pan Q, Sun D, Xu Y, et al. Aspirin Inhibits Cancer Metastasis and Angiogenesis via Targeting Heparanase. Clinical Cancer Research. 2017;23(20):6267-78.

67. Zhang X, Wang Z, Wang Z, Zhang Y, Jia Q, Wu L, et al. Impact of acetylsalicylic acid on tumor angiogenesis and lymphangiogenesis through inhibition of VEGF signaling in a murine sarcoma model. Oncol Rep. 2013;29(5):1907-13.

68. Johnpulle RAN, Pollack MLH, Riemenschneider K, Puzanov I, Sosman JA, Johnson DB. Cyclooxygenase inhibition and response to anti-PD1/L1 in advanced melanoma. Journal of Clinical Oncology. 2016;34(15_suppl):e21023-e.

69. Lawler K, Meade G, O'Sullivan G, Kenny D. Shear stress modulates the interaction of platelet-secreted matrix proteins with tumor cells through the integrin alphavbeta3. Am J Physiol Cell Physiol. 2004;287(5):C1320-7.

70. Roop RP, Naughton MJ, Van Poznak C, Schneider JG, Lammers PE, Pluard TJ, et al. A randomized phase II trial investigating the effect of platelet function inhibition on circulating tumor cells in patients with metastatic breast cancer. Clin Breast Cancer. 2013;13(6):409-15.

71. Bellone M, Calcinotto A, Filipazzi P, De Milito A, Fais S, Rivoltini L. The acidity of the tumor microenvironment is a mechanism of immune escape that can be overcome by proton pump inhibitors. Oncoimmunology. 2013;2(1):e22058-e.

72. Huber V, Camisaschi C, Berzi A, Ferro S, Lugini L, Triulzi T, et al. Cancer acidity: An ultimate frontier of tumor immune escape and a novel target of immunomodulation. Seminars in Cancer Biology. 2017;43:74-89.

73. Calcinotto A, Filipazzi P, Grioni M, Iero M, De Milito A, Ricupito A, et al. Modulation of microenvironment acidity reverses anergy in human and murine tumor-infiltrating T lymphocytes. Cancer Research. 2012;72(11):2746-56.

74. Müller B, Fischer B, Kreutz W. An acidic microenvironment impairs the generation of non-major histocompatibility complex-restricted killer cells. Immunology. 2000;99(3):375-84.

75. Vishvakarma NK, Singh SM. Immunopotentiating effect of proton pump inhibitor pantoprazole in a lymphoma-bearing murine host: Implication in antitumor activation of tumor-associated macrophages. Immunol Lett. 2010;134(1):83-92.

76. Wu X, Hu W, Lu L, Zhao Y, Zhou Y, Xiao Z, et al. Repurposing vitamin D for treatment of human malignancies via targeting tumor microenvironment. Acta Pharmaceutica Sinica B. 2019;9(2):203-19.

77. Pendás-Franco N, García JM, Peña C, Valle N, Pálmer HG, Heinäniemi M, et al. DICKKOPF-4 is induced by TCF/beta-catenin and upregulated in human colon cancer, promotes tumour cell invasion and angiogenesis and is repressed by 1alpha,25-dihydroxyvitamin D3. Oncogene. 2008;27(32):4467-77.

78. Bernardi RJ, Johnson CS, Modzelewski RA, Trump DL. Antiproliferative effects of 1alpha,25-dihydroxyvitamin D(3) and vitamin D analogs on tumor-derived endothelial cells. Endocrinology. 2002;143(7):2508-14.

79. Mantell DJ, Owens PE, Bundred NJ, Mawer EB, Canfield AE. 1 alpha,25-dihydroxyvitamin D(3) inhibits angiogenesis in vitro and in vivo. Circ Res. 2000;87(3):214-20.

80. Sherman MH, Yu RT, Engle DD, Ding N, Atkins AR, Tiriac H, et al. Vitamin D receptor-mediated stromal reprogramming suppresses pancreatitis and enhances pancreatic cancer therapy. Cell. 2014;159(1):80-93.

81. Kong F, Li L, Wang G, Deng X, Li Z, Kong X. VDR signaling inhibits cancer-associated-fibroblasts' release of exosomal miR-10a-5p and limits their supportive effects on pancreatic cancer cells. Gut. 2019;68(5):950-1.

82. Ferrer-Mayorga G, Gómez-López G, Barbáchano A, Fernández-Barral A, Peña C, Pisano DG, et al. Vitamin D receptor expression and associated gene signature in tumour stromal fibroblasts predict clinical outcome in colorectal cancer. Gut. 2017;66(8):1449-62.

83. Min D, Lv XB, Wang X, Zhang B, Meng W, Yu F, et al. Downregulation of miR-302c and miR-520c by 1,25(OH)2D3 treatment enhances the susceptibility of tumour cells to natural killer cell-mediated cytotoxicity. British Journal of Cancer. 2013;109(3):723-30.

84. Lathers DM, Clark JI, Achille NJ, Young MR. Phase 1B study to improve immune responses in head and neck cancer patients using escalating doses of 25-hydroxyvitamin D3. Cancer Immunol Immunother. 2004;53(5):422-30.

85. Kulbersh JS, Day TA, Gillespie MB, Young MRI. 1alpha,25-Dihydroxyvitamin D(3) to skew intratumoral levels of immune inhibitory CD34(+) progenitor cells into dendritic cells. Otolaryngol Head Neck Surg. 2009;140(2):235-40.

86. Zhuravel E, Efanova O, Shestakova T, Glushko N, Mezhuev O, Soldatkina M, et al. Administration of vitamin D3 improves antimetastatic efficacy of cancer vaccine therapy of Lewis lung carcinoma. Exp Oncol. 2010;32(1):33-9.

87. Afsal K, Selvaraj P. Effect of 1,25-dihydroxyvitamin D3 on the expression of mannose receptor, DC-SIGN and autophagy genes in pulmonary tuberculosis. Tuberculosis (Edinb). 2016;99:1-10.

88. Protiva P, Pendyala S, Nelson C, Augenlicht LH, Lipkin M, Holt PR. Calcium and 1,25-dihydroxyvitamin D3 modulate genes of immune and inflammatory pathways in the human colon: a human crossover trial. Am J Clin Nutr. 2016;103(5):1224-31.

89. Kreft B, Brzoska S, Doehn C, Daha MR, Van Der Woude FJ, Sack K. 1,25-dihydroxycholecalciferol enhances the expression of MHC class II antigens and intercellular adhesion molecule-1 by human renal tubular epithelial cells. J Urol. 1996;155(4):1448-53.

90. Paul S, Sa G. Curcumin as an Adjuvant to Cancer Immunotherapy. Frontiers in Oncology. 2021;11:675923.

91. Aggarwal BB, Kumar A, Bharti AC. Anticancer potential of curcumin: preclinical and clinical studies. Anticancer Research. 2003;23(1a):363-98.

92. Shehzad A, Park JW, Lee J, Lee YS. Curcumin induces radiosensitivity of in vitro and in vivo cancer models by modulating pre-mRNA processing factor 4 (Prp4). Chem Biol Interact. 2013;206(2):394-402.

93. Zhao GJ, Lu ZQ, Tang LM, Wu ZS, Wang DW, Zheng JY, et al. Curcumin inhibits suppressive capacity of naturally occurring CD4+CD25+ regulatory T cells in mice in vitro. Int Immunopharmacol. 2012;14(1):99-106.

94. Binion DG, Otterson MF, Rafiee P. Curcumin inhibits VEGF-mediated angiogenesis in human intestinal microvascular endothelial cells through COX-2 and MAPK inhibition. Gut. 2008;57(11):1509-17.

95. Bhandarkar SS, Arbiser JL. Curcumin as an inhibitor of angiogenesis. Adv Exp Med Biol. 2007;595:185-95.

96. Churchill M, Chadburn A, Bilinski RT, Bertagnolli MM. Inhibition of intestinal tumors by curcumin is associated with changes in the intestinal immune cell profile. J Surg Res. 2000;89(2):169-75.

97. Han SS, Chung ST, Robertson DA, Ranjan D, Bondada S. Curcumin causes the growth arrest and apoptosis of B cell lymphoma by downregulation of egr-1, c-myc, bcl-XL, NF-kappa B, and p53. Clin Immunol. 1999;93(2):152-61.

98. Singh S, Aggarwal BB. Activation of transcription factor NF-kappa B is suppressed by curcumin (diferuloylmethane) [corrected]. The Journal of Biological Chemistry. 1995;270(42):24995-5000.

99. Lim SO, Li CW, Xia W, Cha JH, Chan LC, Wu Y, et al. Deubiquitination and Stabilization of PD-L1 by CSN5. Cancer Cell. 2016;30(6):925-39.

100. Hayakawa T, Yaguchi T, Kawakami Y. Enhanced anti-tumor effects of the PD-1 blockade combined with a highly absorptive form of curcumin targeting STAT3. Cancer Science. 2020;111(12):4326-35.
